# One-core neuron deep learning for time series prediction

**DOI:** 10.1093/nsr/nwae441

**Published:** 2024-12-09

**Authors:** Hao Peng, Pei Chen, Na Yang, Kazuyuki Aihara, Rui Liu, Luonan Chen

**Affiliations:** School of Mathematics, South China University of Technology, Guangzhou 510640, China; School of Future Technology, South China University of Technology, Guangzhou 511442, China; School of Mathematics, South China University of Technology, Guangzhou 510640, China; School of Mathematics, South China University of Technology, Guangzhou 510640, China; International Research Center for Neurointelligence, The University of Tokyo Institutes for Advanced Study, The University of Tokyo, Tokyo 113-0033, Japan; School of Mathematics, South China University of Technology, Guangzhou 510640, China; Key Laboratory of Systems Health Science of Zhejiang Province, School of Life Science, Hangzhou Institute for Advanced Study, University of Chinese Academy of Sciences, Chinese Academy of Sciences, Hangzhou 310024, China; Key Laboratory of Systems Biology, Shanghai Institute of Biochemistry and Cell Biology, Center for Excellence in Molecular Cell Science, Chinese Academy of Sciences, Shanghai 200031, China; Guangdong Institute of Intelligence Science and Technology, Zhuhai 519031, China

**Keywords:** spatiotemporal information (STI) transformation, one-core-neuron (OCN), small model, deep learning, time-series prediction, large model

## Abstract

The enormous computational requirements and unsustainable resource consumption associated with massive parameters of large language models and large vision models have given rise to challenging issues. Here, we propose an interpretable ‘small model’ framework characterized by only a single core-neuron, i.e. the one-core-neuron system (OCNS), to significantly reduce the number of parameters while maintaining performance comparable to the existing ‘large models’ in time-series forecasting. With multiple delay feedback designed in this single neuron, our OCNS is able to convert one input feature vector/state into one-dimensional time-series/sequence, which is theoretically ensured to fully represent the states of the observed dynamical system. Leveraging the spatiotemporal information transformation, the OCNS shows excellent and robust performance in forecasting tasks, in particular for short-term high-dimensional systems. The results collectively demonstrate that the proposed OCNS with a single core neuron offers insights into constructing deep learning frameworks with a small model, presenting substantial potential as a new way for achieving efficient deep learning.

## Teaser

The one-core-neuron system (OCNS) is a revolutionary small deep learning model for time-series forecasting, achieving performance comparable to ‘large models’ with a fraction of the parameters.

## INTRODUCTION

Deep learning (DL), particularly through deep neural networks (DNNs), has proven to be highly effective in terms of learning data representations. The DNNs, which are derived from conventional neural networks but significantly outperform their predecessors, excel at capturing complex patterns and features within data. Recently, empowered by DNNs, DL has been widely incorporated into artificial intelligence (AI) infrastructure as an indispensable component and has achieved distinguished performance in a variety of fields, including time-series forecasting [1], computer vision [2], natural language processing [3,4], bioinformatics [5] and recommendation [6]. Nevertheless, the training and deployment expenses of DNNs are substantial since DNNs, especially the large language models (LLMs), e.g. LlaMA [7] and LaMDA [3], typically contain vast numbers of parameters or neurons and require a huge amount of training data to be trained on high-performance computing platforms for extended periods. For example, the LaMDA [3] has up to 137B parameters and needs to be pretrained on 1024 TPU-v3 chips for a total of ∼57.7 days. The massive parameter sizes in the ‘large models’ lead to rapidly growing carbon footprints and unsustainable energy consumption levels, which may result in serious impacts on the environment and climate in the near future [8].

To address the challenges mentioned above, researchers have developed many DNN compression techniques [9,10] to diminish the sizes and complexity levels of DNNs without substantially compromising their performance to an extent. In broad categorization, these methods encompass network pruning [11], quantization [12], low-rank factorization [13] and knowledge distillation approaches [14]. Deep compression [15] and the method detailed in [16] yield significant compression rates in neural networks—ranging from 35× to 49× on basic CNN architectures, like AlexNet [17] and VGG [18], and achieving a maximum of 4.7× on complex ResNets [19]. Despite recent developments in DNN compression, notable challenges persist. For example, identifying a pruning strategy that avoids performance degradation can be daunting [20]. Additionally, quantization introduces quantization errors [9], leading to performance deterioration in specific scenarios. In conclusion, most model-compression methods still require training accurate models with a considerable number of parameters beforehand, and do not address the issue of excessive parameters from a mechanistic or intrinsic perspective. Due to these limitations, challenges remain with regard to bridging the gap between DNNs and various applications, e.g. the Internet of Things (IoT) [21], embedded systems [22] and edge computing [23].

The emergence of machine learning applications [24,25] that incorporate the delay models derived from time-delay dynamical systems [26,27] shed light on the design of new frameworks. This innovation has the potential to intrinsically reduce the number of parameters required to achieve optimal model performance. From a mathematical perspective, continuous time-delay systems possess a noteworthy property wherein their state spaces can be extended to infinite dimensions. In practice, the dynamics of these delay systems with finite dimensions still display the characteristics of short-term memory and high dimensionality [26]. Recently, delay-based reservoir computing [27] has replaced interconnected nodes in the conventional reservoir structure with virtual nonlinear nodes subjected to delayed feedback. Although the prediction accuracy of this method is unsatisfactory, the efficacy of delay-based concepts in reservoir computing has spurred subsequent applications within DNNs. For instance, Fit-DNN [28] was proposed to emulate a full DNN by using only a single neuron with multiple delayed and modulated feedback, which empowers DNNs with sparse connectivity and reduces memory consumption costs. However, this method neither fully explores the temporal dynamics of a single neuron nor provides data representation for dynamical systems.

By assuming that the steady states of a high-dimensional system are constrained on a low-dimensional manifold, which is generally satisfied by dissipative real-world systems, the spatiotemporal information (STI) transformation has theoretically been derived from the delay and non-delay embedding theory [29–32]. The STI transformation equation converts the high-dimensional/spatial data into the temporal information of a latent or target variable, i.e. a high-dimensional state/vector topologically corresponds to a one-dimensional sequence and vice versa. Several methods have recently been developed for predicting short-term time series within the STI transformation framework, with an explicit target variable, e.g. randomly distributed embedding (RDE) [32], the auto-reservoir neural network (ARNN) [33,34] and the spatiotemporal information conversion machine (STICM) [35]. However, none of these methods can simultaneously forecast multivariate states for a dynamical system. If we consider each variable from a high-dimensional system as a target and train the models independently, the computational cost would sharply increase. While existing DL methods generally represent complex high-dimensional data by ‘a latent vector’ (plenty of neurons), the STI equation, in contrast, is able to represent such data by the time series of ‘one latent variable’ rather than a vector, thus providing a new way for DL even with just a single neuron. Notably, time-delayed reservoir computing [36] has recently demonstrated that a few delayed neurons are sufficient to represent the dynamics of a high-dimensional dynamical system, further supporting the feasibility of using a single neuron for deep learning.

**Figure 1.**
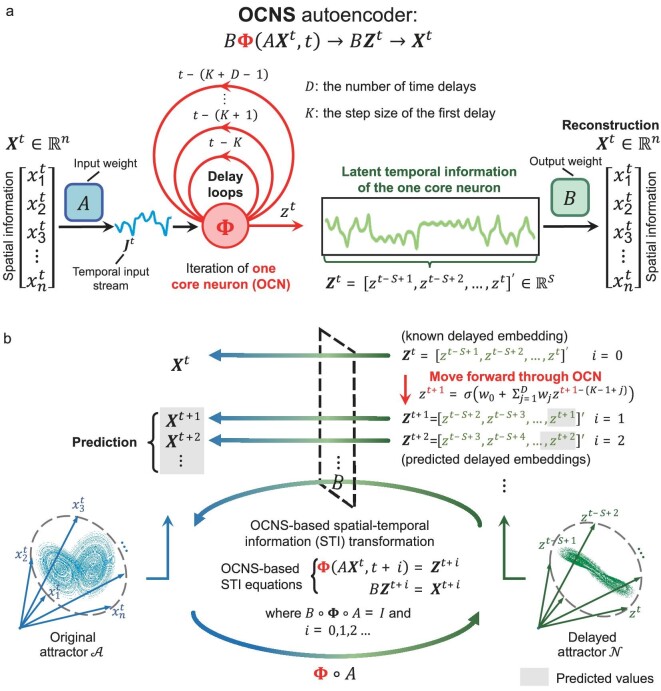
Overview of the OCNS as a ‘small model’ for time-series predicting. (a) The framework of the OCNS is similar to that of an autoencoder. For an observed high-dimensional vector ${{{\boldsymbol{X}}}^t}$, a latent delay vector ${{{\boldsymbol{Z}}}^t}$, comprised of the dynamics of a one-dimensional delay dynamical system ${{z}^t}$, is constructed via the input weight *A* and OCN ${{\bf \Phi }}$ through a delay-embedding scheme. The delay vector ${{{\boldsymbol{Z}}}^t}$ corresponding to time *t* contains the latent temporal information from the delay dynamical system ${{z}^t}$, which can topologically reconstruct all the dynamics of the original system ${{{\boldsymbol{X}}}^t}$. With the output weight *B*, the original spatial information ${{{\boldsymbol{X}}}^t}$ of the original system can be recovered from ${{{\boldsymbol{Z}}}^t}$. The OCN ${{\bf \Phi }}$, which generates a delay dynamical system ${{z}^t}$ with *D* delays feedback in a single neuron-based fashion, is the core of the OCNS. (b) Derived from the solid theoretical foundation of delay dynamical systems [42,43] and the delay embedding theorem [29–31], the information flow of the OCNS is dictated by the OCNS-based STI equations, which encompass both the primary and conjugate STI equations (Eq. 3). Here, we build the delay vector ${{{\boldsymbol{Z}}}^{t + i}} = {{[ {{{z}^{t + i - S + 1}},{{z}^{t + i - S + 2}},\ldots,{{z}^{t + i}}} ]}^{\prime}} \in {{\mathbb{R}}^S} $ at time $t + i$, where $i = 0,1,2\ldots $ and $S $ is the delay-embedding dimension. Specifically, the input weight *A* and OCN ${{\bf \Phi }}$ transform the spatial information in the original attractor $\mathcal{A}$ into the temporal information of the delayed attractor $\mathcal{N}$ corresponding to the primary STI equation, while the conjugate STI equation represents the reconstruction and prediction of the original system constrained on attractor $\mathcal{A}$ from the delayed attractor $\mathcal{N}$ through the output weight *B*. In this way, the OCNS effectively consists of an RNN with one neuron and two linear layers.

In this work, inspired by both the STI transformation equation and machine learning approaches with time-delay models, we introduce a novel small neural network framework (Fig. 1a) called the one-core-neuron-system (OCNS), which is actually a recurrent neural network (RNN [37]) of one neuron with two linear layers. We theoretically demonstrate that the one-core-neuron (OCN) generically is an embedding of the original nonlinear dynamical system (see Theorem 1 of Methods). In addition to the theoretical background, the versatility of the OCNS is demonstrated in tasks such as multivariate time-series forecasting (Fig. 1b) and classification (Fig. S3). In contrast to the existing ‘large models’, such a ‘small model’ OCNS is able both theoretically and computationally to reconstruct the states of the entire high-dimensional dynamical system with a single latent variable, thus enabling data representation learning with only one neuron. Specifically, as a small model, the OCNS achieves comparable or superior performance using on average only 0.035% of the parameters necessary for large-model-based methods [38], while also requiring just 1.16% of the parameters needed by the typical transformer-based approaches [1,39,40]. Intuitively, the OCNS consists of one neuron RNN ${{\bf \Phi }}$ with two linear layers (i.e. two weight matrices *A, B*), simultaneously representing both the primary and conjugate STI equations in an autoencoder form (Fig. 1a). The OCN ${{\bf \Phi }}$ is specifically designed to achieve DL using only a single core neuron. In particular, the input weight *A* and the OCN ${{\bf \Phi }}$ encode the spatial information of original multi-variables (or a state) as the temporal information of a single latent variable (i.e. a one-dimensional time series), while the output weight *B* decodes the latent temporal information as the high-dimensional/spatial information (or state) of the original dynamical system. Based on the STI transformation [32,33,35], the dynamics of the original system can be topologically reconstructed from one latent variable by the OCNS. Once the latent one-dimensional delay dynamical system (derived from the OCN ${{\bf \Phi }}$) is constructed, the latent temporal information can iteratively predict future spatial information through output weight *B* in the conjugate STI equation. In other words, a solid theoretical foundation enables the OCNS to represent the whole states/dynamics of an original system and, furthermore, to forecast multivariate time series in a multistep-ahead manner. Moreover, by fully exploiting the advantages of the one-dimensional delay dynamical system and nonlinear STI transformation, the OCNS only requires a much smaller number of parameters, specifically including *A, B*, and a small subset in the OCN ${{\bf \Phi }}$. And the OCN can almost maintain a small and constant parameter size even when processing a large-scale system. Therefore, the OCNS, as a small model, has a broader range of applications than conventional DL, especially for scenarios with strict restrictions on the number of parameters and specific requirements for learning capacity.

To evaluate the performance of the OCNS, we conduct extensive experiments on the numerical Lorenz datasets [41] and eight real-world datasets. The experimental results numerically demonstrate that the OCNS, as a ‘small model’ with only a single core neuron, can outperform or achieve comparable performance to current forecasting benchmark methods, proving that it can match the performance of ‘large models’ with many more neurons, particularly for short-term high-dimensional systems. Moreover, we extend and generalize the OCNS to image categorization tasks or cross-sectional datasets, and these results also demonstrate the excellent performance of the OCNS. All of the theoretical and computational results show that from a dynamical perspective, the OCNS is powerful and flexible enough to offer a new way to achieve DL with a small model.

## RESULTS

### Dynamics of the OCN represents whole states of a high-dimensional dynamical system in a delay-embedded fashion

Given the high-dimensional spatial input/state ${{{\boldsymbol{X}}}^t} = {{[ {x_1^t,x_2^t,\ldots,x_n^t} ]}^{\prime}} \in {{\mathbb{R}}^n}$ observed from an *n*-dimensional dynamical system at time $t = 0,1,2,\ldots $, we present the main results obtained for two tasks under consideration: dynamical system representation and forecasting. Generally, conventional neural networks utilize many neurons within a multilayer structure to extract high-order features and represent the input data for different tasks. Here, we propose a delayed-dynamic architecture, the OCN ${{\bf \Phi }}$ (Fig. 1a), which can represent the whole high-dimensional data/states by a time-series (delayed vector) ${{{\boldsymbol{Z}}}^t} = {{\bf \Phi }}( {A{{{\boldsymbol{X}}}^t},t} )$ using a single neuron with multiple delays feedback.

To illustrate the main concepts, we consider the single neuron as a one-dimensional system $z( t )$ that evolves in the latent discrete-time *t* and is governed by a delay difference equation (DDE) [24,25,27,28] incorporating multiple temporal delays:


(1) \begin{eqnarray*}
z\left( t \right) &=& \varphi ( {z\left( {t - K} \right),z\left( {t - \left( {K + 1} \right)} \right),\ldots,}\\
&&z\left( {t - \left( {K + D - 1} \right)} \right);t,{{W}_\varphi } ),
\end{eqnarray*}

where $K,\ ( {K + 1} ),\ldots,( {K + D - 1} )$ denote the time delays, *K* is a constant representing the step size of the first delay, *D* is the total number of delays, and ${{W}_\varphi }$ indicates the parameters in the difference function $\varphi ( \cdot )$. Subsequently, we provide a specific form of the function $\varphi ( \cdot )$ employed in this paper only using one neuron:


(2) \begin{eqnarray*}
z\left( t \right) = \sigma \left( {{{w}_0} + \mathop \sum \limits_{j = 1}^D {{w}_j}z\left( {t - \left( {K - 1 + j} \right)} \right)} \right),
\end{eqnarray*}

where $\sigma $ is a nonlinear function, ${{w}_j}, j = 1,2,\ldots,D$ are the weights assigned to each delayed term $z( {t - ( {K - 1 + j} )} )$, and ${{w}_0}$ is a bias term. The parameters ${{W}_\varphi } = {\mathrm{\{ }}{{w}_j}{\mathrm{|}}j = 0,1,\ldots,D\} $ are learned during the training process. Figure 1a shows how the OCN architecture ${{\bf \Phi }}$ processes the iterative acquisition of the inner delayed dynamics for ${{z}^t}$. Depending on the number of delay terms *D*, the OCN ${{\bf \Phi }}$ is able to capture the complex dynamics of the high-dimensional data. For convenience of description, we denote $z( t )$ as ${{z}^t}$ in the following context. To extend the processing capability of the OCN ${{\bf \Phi }}$ to spatial information, the input state ${{{\boldsymbol{X}}}^t}$ of any dimension *n* is multiplied by a mask (the input weight $A \in {{\mathbb{R}}^{L \times n}}$), resulting in a temporal input stream ${{{\boldsymbol{J}}}^t} = A{{{\boldsymbol{X}}}^t} \in {{\mathbb{R}}^L}$ (Fig. 1a), which is then fed into the OCN ${{\bf \Phi }}$ as the initial temporal states. For representing the whole states of a high-dimensional dynamical system ${{{\boldsymbol{X}}}^t}$, we define a delay vector ${{{\boldsymbol{Z}}}^t} = {{\bf \Phi }}( {{{{\boldsymbol{J}}}^t},t} ) = {{\bf \Phi }}( {A{{{\boldsymbol{X}}}^t},t} ) = {{[ {{{z}^{t - S + 1}},{{z}^{t - S + 2}},\ldots,{{z}^t}} ]}^{\prime}} \in {{\mathbb{R}}^S}$ which is presented in Fig. 1a, where *S* is the delay-embedding dimension. Therefore, the input state ${{{\boldsymbol{X}}}^t}$ at any time *t* is theoretically (see Theorem 1 of Methods) represented by the delayed dynamics of the single-variable system ${{z}^t}$. We provide a comprehensive workflow of the OCN ${{\bf \Phi }}$ utilizing the difference function $\varphi ( \cdot )$ in the second section of ‘Methods’.

The information flow within the OCN ${{\bf \Phi }}$ resembles that of a general RNN [37], i.e. the dynamics of the system ${{z}^t}$ results from the iterative propagation of feedback signals through the same delay dynamic function $\varphi $. Note that the scale of the parameters ${{W}_\varphi }$ in the OCN remains fixed and does not vary with the length of the output time series. In such a way, the required parameters for models are significantly reduced, while the complexity of the dynamics in ${{z}^t}$ is guaranteed by the delayed feedback in the delay dynamical system [42,44,45], which has profound implications on the representation performance of the OCN. In summary, the main idea of the OCN is that the features extracted from the spatial vectors are latent temporal sequentializations (time series) that derived from a one-dimensional delay dynamical system ${{z}^t}$. In theory, the OCN with the delay-embedding scheme can fully represent the states of a dynamical system (see Theorem 1 of Methods). In contrast to existing DL methods, which generally represent high-dimensional data by a latent vector, the OCNS represents such data by a latent variable (time series), thus paving a new way for DL even with a single neuron.

To further enhance the representational capacity of the single neuron in the OCN ${{\bf \Phi }}$, the concept of ‘sub-time’ can be theoretically incorporated within the OCN framework. Additionally, the framework of the difference function $\varphi ( \cdot )$ implemented by a single neuron (Eq. 2) can be substituted with a small neural network having a one-dimensional output for improving the representational capability of the OCN ${{\bf \Phi }}$ (see Section S1).

### The OCNS framework with STI transformation for time-series prediction

To further illustrate how the OCNS achieves multivariate time-series forecasting, Fig. 1b and Fig. S1 present the main conceptual structure for topologically reconstructing the original system via a latent one-dimensional delay dynamical system ${{z}^t}$ and further forecasting the original system with the OCNS-based STI equations (Eq. 3). For each observed high-dimensional/spatial state ${{{\boldsymbol{X}}}^t} = {{[ {x_1^t,x_2^t,\ldots,x_n^t} ]}^{\prime}}$ containing *n* variables with time superscript *t*, a corresponding delay (temporal) vector ${{{\boldsymbol{Z}}}^t} = {{[ {{{z}^{t - S + 1}},{{z}^{t - S + 2}},\ldots,{{z}^t}} ]}^{\prime}} \in {{\mathbb{R}}^S}$ is constructed from the latent one-dimensional delay dynamical system ${{z}^t}$, *S* denotes the delay-embedding dimension, and ‘ $^{\prime}$ ’ is the transpose of a vector. Based on the delay embedding theory [29,30] and its generalized forms [29,46], the dynamics of the original system can be topologically reconstructed from a delay-embedding scheme if $S > 2d > 0$, where *d* is the Minkowski dimension of the attractor (see Methods). Combining the OCN ${{\bf \Phi }}$ and STI transformation, we introduce an OCNS framework which performs multivariate forecasting for the original system based on both the primary and conjugate STI equations (Fig. 1b); i.e. the OCNS can be represented by the following OCNS-based STI equations:


(3) \begin{eqnarray*}
\left\{ {\begin{array}{@{}*{1}{c}@{}} {\Phi \left( {A{{{\boldsymbol{X}}}^t},t + i} \right) = {{{\boldsymbol{Z}}}^{t + i}}}\\ {B{{{\boldsymbol{Z}}}^{t + i}} = {{{\boldsymbol{X}}}^{t + i}}} \end{array}} \right.,\quad i = 0,1,2,\ldots,
\end{eqnarray*}

where *i* represents the prediction step index, $A \in {{\mathbb{R}}^{L \times n}}$ and $B \in {{\mathbb{R}}^{n \times S}}$ are the input and output weight matrices, respectively. Indeed, the OCNS-based STI equations benefit from the principles of delay dynamical systems [42,47] and the delay embedding theorem [29–31]. Specifically, the first formula in Eq. 3 is the primary equation, which depicts that the dynamics of the observed high-dimensional data ${{{\boldsymbol{X}}}^t}$ are captured within the one-dimensional delay dynamical system ${{z}^t}$ produced by the OCN ${{\bf \Phi }}$. The second formula in Eq. 3 is the conjugate equation, which shows that the dynamics of the original system ${{{\boldsymbol{X}}}^t}$ can be reconstructed from the latent temporal information ${{{\boldsymbol{Z}}}^t}$ through output weight *B*. The STI equations Eq. 3) hold when some generic conditions are satisfied according to the delay embedding theory [29,30].

**Figure 2.**
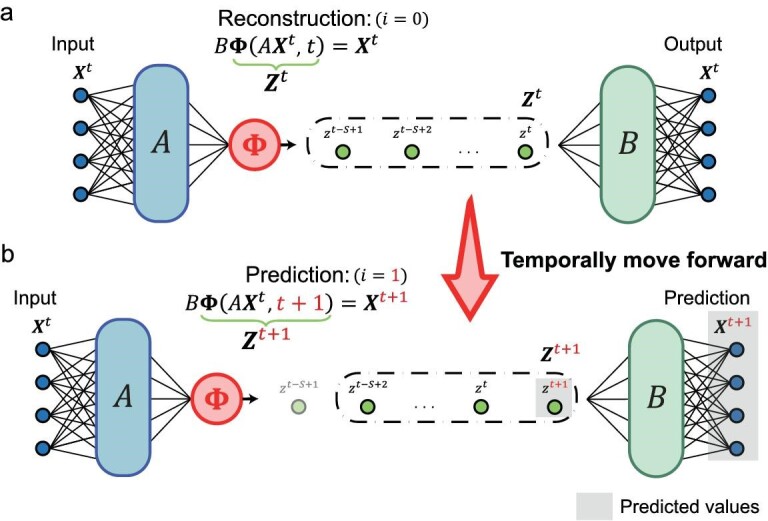
Diagram of our OCNS schema to make one-step forward prediction. (a) Our OCNS is based on an autoencoder form, which can extract intrinsic temporal information ${{{\boldsymbol{Z}}}^t} = {{\bf \Phi }}( {A{{{\boldsymbol{X}}}^t},t} )$ within the delay dynamical system ${{z}^t}$ from the original dynamical system ${{{\boldsymbol{X}}}^t}$ and decode the temporal information to recover ${{{\boldsymbol{X}}}^t} = B{{{\boldsymbol{Z}}}^t}$. (b) Consequently, the prediction form ${{{\boldsymbol{X}}}^{t + 1}} = B{{{\boldsymbol{Z}}}^{t + 1}}$ of the OCNS can be achieved by rolling the one-dimensional delay dynamical system forward to generate the temporal information at time point $t + 1$ (${{{\boldsymbol{Z}}}^{t + 1}} = {{\bf \Phi }}( {A{{{\boldsymbol{X}}}^t},t + 1} )$).

To further elucidate the prediction steps of the OCNS, in Fig. 2, we illustrate how the OCNS performs a one-step prediction based on ${{{\boldsymbol{X}}}^t}$. First, as shown in Fig. 2a, the OCNS with $i = 0$ operates similarly to an autoencoder. In this step, the spatial vector of ${{{\boldsymbol{X}}}^t}$ is transformed into ${{{\boldsymbol{Z}}}^t}$, a time series of the one-dimensional variable *z* through the input weight *A* and the OCN ${{\bf \Phi }}$; then, the output weight *B* reconstructs the original spatial vector from the latent time series ${{{\boldsymbol{Z}}}^t}$. Subsequently, Fig. 2b illustrates the one-step prediction ($i = 1$) process within the OCNS framework. In practice, the latent one-dimensional system of ${{z}^t}$ evolves automatically based on historical states, thereby yielding the latent future temporal state ${{z}^{t + 1}}$ for the next time stamp $t + 1$. And then the corresponding delay vector ${{{\boldsymbol{Z}}}^{t + 1}} = {{\bf \Phi }}( {A{{{\boldsymbol{X}}}^t},t + 1} ) = {{[ {{{z}^{t - S + 2}},{{z}^{t - S + 3}},\ldots,{{z}^{t + 1}}} ]}^{\prime}}$ can be constructed to further obtain the states ${{{\boldsymbol{X}}}^{t + 1}}$ of the original system through output weight *B* (details in ‘Methods’). Based on this iterative forward-propagation within the system ${{z}^t}$, the proposed OCNS can predict the future states of the original system ${{{\boldsymbol{X}}}^t}$.

Specifically, let *m* and *l* denote the lengths of the known/observed time series and the desirable horizon to be predicted ahead of the current time stamp, respectively. According to the OCNS-based STI equations (Eq. 3), we can transform the observed high-dimensional time series $[ {{{{\boldsymbol{X}}}^{t + 1}},{{{\boldsymbol{X}}}^{t + 2}},\ldots,{{{\boldsymbol{X}}}^{t + m}}} ]$ into a delay-embedding matrix of the latent delay dynamical system ${{z}^t}$ as follows:


(4) \begin{eqnarray*}
&&\left[ {{{{\boldsymbol{Z}}}^{t + 1}},{{{\boldsymbol{Z}}}^{t + 2}},\ldots,{{{\boldsymbol{Z}}}^{t + m}}} \right]\\
&&\quad = {{\left[ {\begin{array}{@{}*{4}{c}@{}} {{{z}^{t - S + 2}}}&\quad {{{z}^{t - S + 3}}}&\quad \cdots &\quad {{{z}^{t + 1}}}\\ {{{z}^{t - S + 3}}}&\quad {{{z}^{t - S + 4}}}&\quad \cdots &\quad {{{z}^{t + 2}}}\\ \vdots &\quad \vdots &\quad \ddots &\quad \vdots \\ {{{z}^{t - S + m + 1}}}&\quad {{{z}^{t - S + m + 2}}}&\quad \cdots &\quad {{{z}^{t + m}}} \end{array}} \right]}^{\prime}}.\\
\end{eqnarray*}

Once the OCN ${{\bf \Phi }}$ is determined, the latent delay dynamical system ${{z}^t}$ encapsulates the inherent dynamics of the original system, and it can evolve the future states in the latent space over *l* time steps from the known latent dynamics. Thus, the future multivariate states $[ {{{{\boldsymbol{X}}}^{t + m + 1}},{{{\boldsymbol{X}}}^{t + m + 2}},\ldots,{{{\boldsymbol{X}}}^{t + m + l}}} ]$ of the original system can be obtained by multiplying the output weight *B* with the delay-embedding matrix (STI conjugate equation in Eq. 3):


(5) \begin{eqnarray*}
&&\left[ {{{{\boldsymbol{X}}}^{t + m + 1}},{{{\boldsymbol{X}}}^{t + m + 2}},\ldots,{{{\boldsymbol{X}}}^{t + m + l}}} \right]\\
&&= B\left[ {{{{\boldsymbol{Z}}}^{t + m + 1}},{{{\boldsymbol{Z}}}^{t + m + 2}},\ldots,{{{\boldsymbol{Z}}}^{t + m + l}}} \right]\\
&& = B{{\left[ {\begin{array}{@{}*{4}{c}@{}} {{{z}^{t - S + m + 2}}}&\,\,\,\, {{{z}^{t - S + m + 3}}}&\,\,\,\, \cdots &\,\,\,\, {{{z}^{t + m + 1}}}\\ {{{z}^{t - S + m + 3}}}&\,\,\,\, {{{z}^{t - S + m + 4}}}&\,\,\,\, \cdots &\,\,\,\, {{{z}^{t + m + 2}}}\\ \vdots &\,\,\,\, \vdots &\,\,\,\, \ddots &\,\,\,\, \vdots \\ {{{z}^{t - S + m + l + 1}}}&\,\,\,\, {{{z}^{t - S + m + l + 2}}}&\,\,\,\, \cdots &\,\,\,\, {{{z}^{t + m + l}}} \end{array}} \right]}^{\prime}},\\
\end{eqnarray*}

where the future latent dynamic states $\{ {{{z}^{t + m + 1}},{{z}^{t + m + 2}},\ldots,{{z}^{t + m + l}}} \}$ are located under the shadow area. Here, we limit the prediction length of the OCNS to *l* (Eq. 5), even though the forecasting horizon can theoretically be infinite. The detailed pseudocodes of OCNS are provided in Section S13.

It is evident that utilizing the primary equation (Eq. 3) to encode the embedding of the original dynamical system is theoretically guaranteed (see Methods). However, decoding (the conjugate equation) the embedding back to the original system's spatial information is relatively challenging. Here, we use the linear matrix *B* to decode in order to maintain a low computational load for the model. Employing a stronger decoder instead of the linear output weight *B* can further enhance the performance of OCNS (for detailed information, refer to Section S1).

### Multivariable time-series prediction on a Lorenz system

To verify the mechanism and the basic idea of the OCNS framework, we first apply the OCNS to perform multivariate forecasting tasks on synthetic time-series datasets with different noise conditions, which are generated by a 9-dimensional coupled Lorenz model [41]:


(6) \begin{eqnarray*}
{\boldsymbol{\dot{X}}}\left( t \right) = G\left( {{\boldsymbol{X}}\left( t \right);Q} \right),
\end{eqnarray*}

where *Q* is a parameter vector of the function set $G( \cdot )\ $and ${\boldsymbol{X}}( t ) = [ {x_1^t,x_2^t,\ldots,x_9^t} ]^{\prime}$ denotes the system state at time *t*. The details of the Lorenz system (Eq. 6) are presented in Section S3. For this Lorenz system, the OCNS uses a time series of $20$ steps (i.e. input steps $m = 20$) to forecast the future states of all 9 variables in the next $l = 8$ time points. Building on the success of the OCNS, we developed an enhanced variant, OCNS^+^, incorporating several improvements such as introducing a neural network module with one-dimensional output rather than a single neuron, integrating ‘sub-time’, and utilizing a more powerful decoder. This enhanced version has also been validated in experiments, demonstrating its effectiveness in handling more complex scenarios.

**Figure 3.**
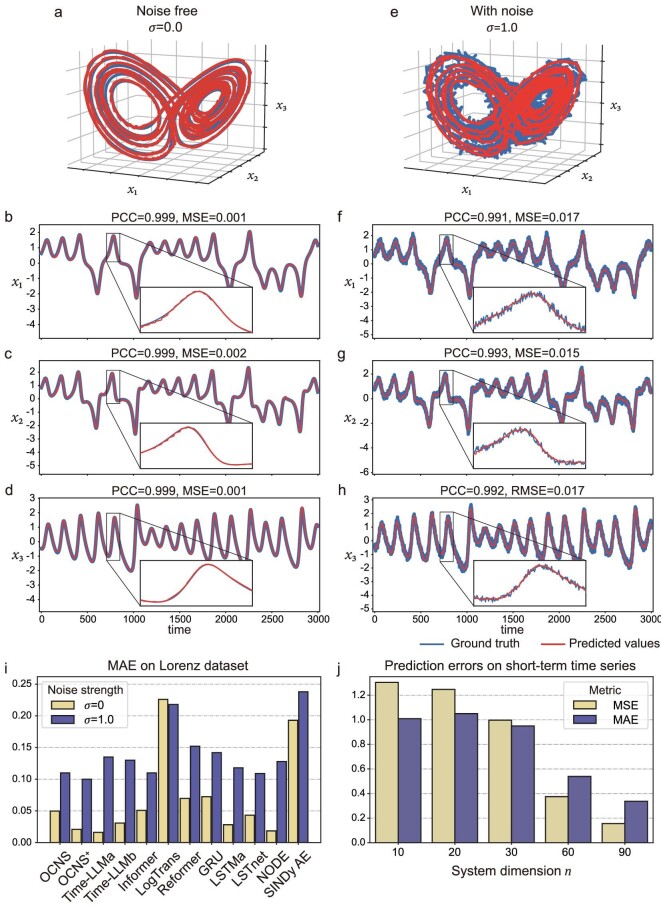
Forecasting a Lorenz system using the OCNS. Ground truth (blue) and predicted (red) attractors of the Lorenz system under the noise-free setting (a) and a noise strength of $\sigma = 1.0$ (e). (b)–(d) Overlayed long-term forecasting results produced by the OCNS on the test dataset for the first three variables ${{x}_1},{{x}_2},{{x}_3}$ without noise. (f)–(h) When the noise strength $\sigma = 1.0$, the OCNS can still provide better prediction results on the test dataset. (i) Illustrates the MAE values of the OCNS, OCNS^+^ and comparative methods when applied to the Lorenz system under a noise-free condition and a condition with noise strength $\sigma = 1.0$. (j) The prediction performance of OCNS on short-term time series obtained from the Lorenz system with different dimensions.

**Noise-free situation.** First, the OCNS is applied to a noise-free Lorenz system. Figure 3a illustrates the similarity of the predicted values (red line) and ground truth (blue line) of Lorenz strange attractors composed of the first three variables, i.e. ${{x}_1},{{x}_2},{{x}_3}$. As shown in Fig. 3b–d, the long-term forecasting results of the OCNS achieve high accuracy, with mean squared errors (MSEs) of 0.001, 0.002, 0.001 and Pearson correlation coefficients (PCCs) of 0.999, 0.999, 0.999 for variables ${{x}_1},{{x}_2},{{x}_3}$, respectively. The performance of the OCNS in terms of forecasting the other six variables of the Lorenz system is shown in Fig. S6.

**Additive noise situation.** Second, we demonstrate the performance of the OCNS on the Lorenz system with additive white noise (noise strengths of $\sigma = 0.5$ and $\sigma = 1.0$). The forecasting horizon is the same as that applied in the noise-free situation. For a noise strength of $\sigma = 0.5$, the OCNS demonstrates robustness performance on the Lorenz system, as illustrated in Figs S5 and S7. Even in the presence of a higher level of added noise ($\sigma = 1.0$), the OCNS continues to exhibit discernible competence in terms of capturing the intricate dynamics of the Lorenz strange attractor, as evidenced in (Fig. 3e), albeit with a slight decline in performance compared to that achieved in the noise-free condition. Specifically, the MSEs slightly increase to 0.017, 0.015 and 0.017 for the variables ${{x}_1},{{x}_2},{{x}_3}$, respectively. Furthermore, the PCCs exhibit modest decreases, with values of 0.991, 0.993 and 0.992 for ${{x}_1},{{x}_2},{{x}_3}$, respectively. Figure 3f–h depict the corresponding outcomes obtained for each of them with the system perturbated by noises. Similar to the noise-free case, the prediction results of the remaining six variables by the OCNS are presented in Fig. S8. Moreover, the OCNS shows superior performance in effectively predicting short-term high-dimension data. As depicted in Fig. 3j, despite only 400 time points in total, the OCNS makes accurate predictions by efficiently utilizing spatial information through STI transformation. The details of the comparison, showing that the OCNS outperforms the majority of other methods in most scenarios, are provided in Section S6.

**Comparison with baseline methods.** Furthermore, we evaluate the performance of our OCNS and OCNS^+^ by comparing it with one LLM-based method, i.e. Time-LLM [38], and eight contemporary DL prediction methods: Informer [1], LogTrans [40], Reformer [39], GRU [48], LSTMa [49], LSTNet [50], Neural ODE (NODE) [51] and SINDy autoencoder (SINDy AE) [52]. For Time-LLM, we select two backbone LLMs in the experiments: GPT-2 [53] (Time-LLM^a^) and LlaMA-7B [7] (Time-LLM^b^). We provide a concise overview of the functionalities of these baseline methods in Section S9. In line with the approach taken in the Informer study [1], we employ three evaluation metrics to assess the performance of our methodology, including PCC, MSE $= \frac{1}{l}\mathop \sum \nolimits_{t = 1}^l {{( {x_i^t - \hat{x}_i^t} )}^2}$, and mean absolute error (MAE $= \frac{1}{l}\mathop \sum \nolimits_{t = 1}^l | {x_i^t - \hat{x}_i^t} |$), where $\hat{x}_i^t$ denotes the forecasting value of the *i*-th variable at time stamp *t* and $x_i^t$ is the ground truth. Figure 3i and Table S7 present an overview of the multivariate forecasting performances conducted by the OCNS, OCNS^+^ and ten baseline methods on the Lorenz system (Eq. 6). The comparisons encompass three distinct cases: (i) a noise-free case, (ii) a perturbed case with noise strength $\sigma = 0.5$ and (iii) a perturbed case with noise strength $\sigma = 1.0$. Specifically, in noise-free condition ($\sigma = 0$), OCNS and OCNS^+^ exhibit slightly higher prediction errors compared to LLM-based and NODE methods. However, OCNS and OCNS^+^ demonstrate notable robustness in the presence of noise ($\sigma = 0.5\ $and $\sigma = 1$).

### Time-series prediction by the OCNS on real-world datasets

The significance of time-series forecasting cannot be overstated, as it plays a crucial role in various real-world applications, including weather forecasting, electricity consumption planning, etc. In this study, we apply the OCNS to eight real-world datasets from diverse fields, including three electricity transformer temperature (ETT) datasets [1], an electricity consumption load (ECL) dataset [54], a large server machine dataset (SMD) [55], one weather dataset (WTH_1_) from the United States (https://www.ncei.noaa.gov/data/local-climatological-data/) and a large weather dataset (WTH_2_) from Beutenberg, Germany (https://www.bgc-jena.mpg.de/wetter/). For all datasets except ECL, we set the known length (*m*) to 48 (the observed data length) and the forecasting length (*l*) to 24 (the predicted data length). For the ECL dataset, the known length (*m*) is set to 96, and the forecasting length (*l*) is set to 48. The detailed description of each dataset is given in Section S4 and Table S5.

**Figure 4.**
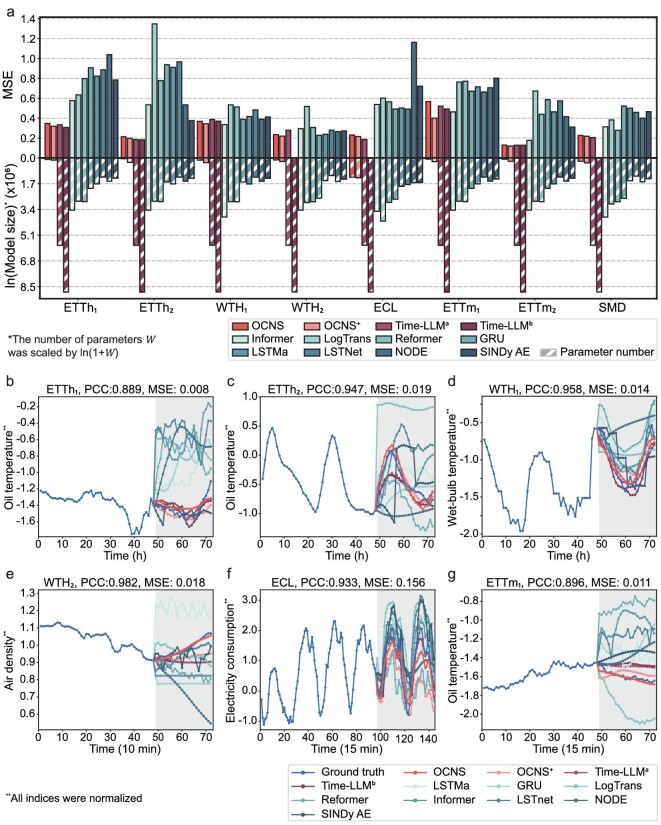
Forecasting results obtained on real-world datasets using the OCNS. (a) We evaluate the MSEs and numbers of model parameters required by the OCNS, OCNS^+^ and the ten comparative methods on all real-world datasets. The results evaluated by the MAE are shown in Fig. S9. The MSEs of Time-LLM^b^ on WTH_2_, ECL and SMD datasets could not be obtained because the training time exceeded the 24-hour time limit. The forecasting results of the OCNS are visually compared with those of the other ten prediction methods on the ETTh_1_ (b), ETTh_2_ (c), WTH_1_ (d), WTH_2_ (e), ECL (f) and ETTm_1_ (g) datasets.

A bidirectional bar chart in Fig. 4a presents the comparative analysis among the OCNS, OCNS^+^ and the ten baseline methods. The results depict that the OCNS achieves comparable performance, while the OCNS^+^ outperforms all baselines in most cases. In particular, the upper part of Fig. 4a shows the MSE of forecasting, while the lower part elucidates the number of parameters required by each approach across various real-world datasets. Notably, all parameter counts (*W*) are subjected to a logarithmic transformation, specifically $\ln ( {1 + W} )$, enhancing the visual clarity and intuitiveness of the presentation. As shown in Fig. 4a, the proposed OCNS demonstrates exceptional efficiency by achieving superior or comparable prediction performance with a minimal parameter count. Meanwhile, OCNS^+^ achieves even better performance at the cost of slightly increasing the parameter number. The dynamic architectural design of the OCNS results in remarkable efficiency, attaining optimal predictive outcomes while operating with a significantly reduced parameter footprint. Further analysis on model efficiency, including training time and memory usage, can be found in Section S10. Figure S9 offers a detailed analysis in terms of the MAE metric. Complementarily, a detailed exposition of the quantitative metrics produced by both the proposed OCNS, OCNS^+^ and the ten comparative methods is presented in Table S7. Figure 4b–g exhibit forecasting examples from six real-world datasets, showcasing both the ground truth observations and the corresponding forecasting outcomes derived from various methods. For all the real-world datasets, *z*-score standardization is employed such that the mean and standard deviation of a variable are 0 and 1, respectively.

**ETT datasets.** The ETT data, collected from two separate counties in China between July 2016 and July 2018 [1], were used to generate four datasets: ETTh_1_ and ETTh_2_ at the 1-hour level, and ETTm_1_ and ETTm_2_ at the 15-minute level. Here, we examine oil temperature forecasting as an illustrative example across ETT datasets (Fig. 4b, c, and g), from which we can observe that the OCNS is able to precisely capture and forecast the intricate dynamical evolution process of oil temperature with a low MSE and a high PCC.

**Weather datasets.** The climate system, as a typically complex and chaotic system, presents challenges for weather forecasting and climate modeling due to its recurrent large-scale configurations intertwined with essential physical properties. In this work, we introduce two weather datasets, namely, WTH_1_ and WTH_2_, recorded in the United States and Germany, respectively, to validate the forecasting performance of the OCNS. We proceed to visually represent the wet-bulb temperature results obtained on the WTH_1_ (Fig. 4d) and air density forecasting results obtained on WTH_2_ (Fig. 4e) datasets, respectively. Both forecasting results distinctly demonstrate the superior performance exhibited by the OCNS in contrast with the other methods. In particular, the MSE and PCC for WTH_1_ (Fig. 4d) stand at 0.014 and 0.958, respectively. For WTH_2_ (Fig. 4e), the corresponding values are 0.018 for the MSE and 0.982 for the PCC.

**ECL dataset.** The original ECL dataset consists of electricity consumption data recorded every 15 minutes (Kwh) for a total of $n = 321$ clients [54]. The forecasting results of the 12 distinct methods, with a focus on the electricity consumed by the 321st client, are presented as demonstrative instances (Fig. 4f). Evidently, the predicted electricity consumption values generated by the OCNS exhibit remarkable consistency with the ground truth values yielding an ${\mathrm{MSE}} = 0.156$, surpassing the predictive performance of the other ten baseline methods.

Additional illustrative examples are provided in Figs S10–S17.

It is clear that the OCNS and OCNS^+^ perform well in prediction tasks across real-world datasets of the electrical engineering and meteorology fields. The simple architecture and underlying mechanism of the OCNS allows it to achieve adequate representation performance with substantially fewer parameters. For example, as shown in Fig. 4a and Table S8, the OCNS achieves the highest efficiency with a parameter size as little as 0.00017% of that required by the Time-LLM^b^, showcasing the effectiveness of the OCNS in achieving accurate predictions with a minimal parameter footprint. Furthermore, to examine the performance of each method in the case with the same parameter scale, experiments are conducted by the competing methods with reduced parameters to match the scale of the OCNS. In Table S9, we present the quantitative measurements yielded by the proposed OCNS and the parameter-reduced counterparts of the eight other baseline methods (denoted by asterisks). Note that the Time-LLM method is not included in this experiment since its reliance on pre-trained LLMs prevents reducing its parameter count to the scale of OCNS. As shown, all the parameter-reduced variants exhibit markedly inferior performance to that of the corresponding original approaches (the increasing percentages of MSE and MAE over the original methods are shown in brackets following each metric value in Table S9). These eight parameter-reduced counterparts all show high MAEs and MSEs, with average increases of ∼41% and 62%, respectively, compared to their original forms. Notably, when considering methods with equivalent parameter magnitudes, the proposed OCNS distinctly shows superior performance across all evaluation metrics in various real-world datasets.

In summary, all these results demonstrate the ability of OCNS to capture the inherent dynamical behaviors of complex real-world systems through its unique OCN architecture, further solidifying its effectiveness and potency as a small model in multivariate time-series forecasting tasks.

### Classification by deep learning of the OCN for cross-sectional data

In addition to the suitability for forecasting dynamical systems, the OCNS can also be easily extended to image classification tasks or non-time series datasets. In the context of image classification, the OCNS has a role analogous to that of an autoencoder, encoding and decoding an image by compressing it into a temporal sequence of a single latent variable, thereby presenting a novel representation approach. To demonstrate the computational capabilities of the OCNS, we compare it with two baseline classification approaches, the vanilla multilayer perceptron (MLP) and Fit-DNN [28], which realizes a DNN via a single neuron with feedback-modulated delay loops. The evaluation encompasses five classification datasets that are identical to those employed in the Fit-DNN approach, including MNIST [17], Fashion-MNIST [56], CIFAR-10, CIFAR-100 with coarse class labels [57], and the cropped version of SVHN [58]. For tasks of image classification, we release the constraint imposed on the original OCNS, wherein the OCN was required to adhere to the dynamics of delay embedding. Specifically, the pseudo-temporal feature sequence of length *N* derived from the OCN module for each image is directly employed for representation and classification purposes. This adjustment is implemented in response to the absence of inter-image dynamics between distinct images, a major difference from the scenarios of multivariate time-series forecasting. Meanwhile, reconstruction for the input image is retained as a form of regularization (Table S3). A framework overview and more details on the implementation of the OCNS in classification tasks are provided in Section S7 and Fig. S4.

We also investigate the influence of the total length of latent time points, *N*, used in the OCN module, by adopting the same strategy as in Fit-DNN [28]. As demonstrated in Table S10, an increase in *N* distinctly correlates with enhanced classification accuracy across the five benchmark datasets. Clearly, our OCNS outperforms Fit-DNN and the vanilla MLP on all datasets. In the cases involving the simpler datasets (MNIST and Fashion-MNIST), all three methods achieve comparable and satisfactory accuracy. However, for more challenging datasets such as CIFAR-10, CIFAR-100 with coarse class labels, and the cropped version of SVHN, Fit-DNN significantly lags behind the performances of the OCNS and MLP. Specifically, the OCNS beats Fit-DNN by margins of 12.10%, 12.92% and 13.08% on CIFAR-10, CIFAR-100 and SVHN, respectively. The details of data augmentation are provided in Section S8. Although the OCNS does not surpass the current SOTA transformer-based or CNN-based methods [59,60] in terms of classification performance, it presents a highly innovative framework and methodology. Additionally, the OCNS exhibits great potential for more effective integration with deep learning in the future, promising profound implications for an efficient AI infrastructure.

## DISCUSSION

In this study, we proposed a novel deep learning framework called the OCNS. Unlike existing deep learning models that typically contain many neurons and layers to represent complex high-dimensional data using a latent vector, the OCNS, as a small model, comprises a single core neuron with merely two linear layers to represent high-dimensional time-series data using a latent variable (rather than a vector). One prominent advantage is that the OCNS can fully exploit the complexity of the dynamics in the delay system, ensuring high capacity. Meanwhile, it significantly reduces the parameter scale of a deep learning model, requiring only a fraction (e.g. 0.00017%) of the parameters in LLM-based methods. Based on the delay embedding theorem, the OCNS is proposed to represent the whole states of a dynamical system even with only a one-dimensional latent variable, and we derive the OCNS-based STI equations (Eq. (3)), i.e. one high-dimensional state corresponds to one-dimensional sequence and vice versa. Specifically, the OCNS is an autoencoder-based framework that can solve both primary and conjugate OCNS-based STI equations. From this perspective, the input weight *A* and the OCN ${{\bf \Phi }}$ collaboratively serve as the encoder, transforming the spatial information of dynamical systems into temporal dynamics, while the output weight *B* functions as the decoder, reconstructing the original system from the latent time series. In other words, the STI equations enable the OCNS, as a small model, to create a latent one-dimensional delay dynamical system that can encode and topologically reconstruct the original dynamical system. Generally, explicitly reconstructing the original dynamical system ${{{\boldsymbol{X}}}^t}\ $from the nonlinear observable ${{{\boldsymbol{Z}}}^t}$ (temporal dynamics) requires a nonlinear function *f*, i.e. ${{{\boldsymbol{X}}}^t} = f( {{{{\boldsymbol{Z}}}^t}} )$. Using a linear matrix *B* for decoding raises a concern that it might limit the model's ability to decode highly nonlinear information. However, our empirical evaluations show that the nonlinear OCN ${{\bf \Phi }}$ effectively compensates for this limitation, thus producing high-quality forecasting results, in particular for short-term high-dimensional data. One candidate for the conjugate OCNS-based STI equation can be a multilayer nonlinear neural network, as demonstrated by the superior performance of OCNS^+^. Depending on the task complexity, it is feasible to choose a larger decoder, or even some larger models.

Our approach differs from a recent, promising time-delayed reservoir computing (RC) model [36], which introduces time delays only in the output layer. The delay mechanism allows the delayed RC to match the performance of larger RCs while reducing the reservoir's physical size, providing flexible memory capacity to handle more complex dynamic reconstruction tasks than a standard RC of the same size. While these two methods share some similarities, such as both featuring evolution dynamics and a linear time-delayed readout, OCNS uniquely combines the strengths of autonomous evolution (delay dynamical systems) and delay embedding schemes. Additionally, OCNS trains parameters for both linear decoding and nonlinear forward propagation, enhancing its ability to capture the nonlinear characteristics of the original systems. A detailed comparison is provided in Section S14.

From the perspective of reusing small models systematically and thoroughly, the recently proposed learnware paradigm [61–63] addresses challenges that remain unsolved by prevailing singular large models, such as the lack of training data and skills, catastrophic forgetting, and the complexities of continual learning. Simultaneously, learnware obviates the necessity for users to construct a model from scratch. Although the current learnware paradigm is in a preliminary stage and poses numerous issues for future exploration, such a concept enables existing trained small models to be competent in most tasks, thus effectively reducing carbon emissions by minimizing redundant model deployments. In contrast to the learnware paradigm, we propose a minimal and concise OCNS that utilizes an OCN to represent the entire dynamical system, thereby inherently ensuring good-enough performance for small models. At the same time, the concept of the learnware paradigm offers profound inspiration for the future design of a more refined and generalized OCNS framework. For instance, we envision adopting a divide-and-conquer approach by incorporating multiple interacting OCNs to represent an exceedingly complex dynamical system, with each OCN responsible for representing a portion of the system.

Several ongoing challenges and potential research directions remain and motivate future work. First, the integration of the OCNS with deep learning is limited to the MLP architecture so far, while deep learning includes several other foundational network models, such as CNNs and transformers. Designing a more general delay system architecture based on these frameworks might unlock more groundbreaking methods. Besides, it is still critical to further explore efficient gradient computation methods that are tailored for delay dynamical systems. In conclusion, in contrast to the existing ‘large models’, the proposed OCNS paves a new way for achieving efficient deep learning and AI through a small model framework, offering significant potential for a wide range of real-world applications.

## METHODS

Detailed methods are given in the online Supplementary Materials.

## Supplementary Material

nwae441_Supplemental_File

## Data Availability

All data needed to validate the conclusions are present in the paper and/or the Supplementary Materials. The code used in this study is available at https://github.com/Peng154/OCN.
