# Supplementary material for: One-core neuron deep learning for time series prediction
Source: Natl Sci Rev. 2024 Dec 9;12(2):nwae441. doi: 10.1093/nsr/nwae441 (PMC11737406; doi:10.1093/nsr/nwae441)
Supplement: nwae441_Supplemental_File [file nwae441_supplemental_file.pdf]

Supplementary materials for

One-core neuron deep learning for time series prediction

Hao Peng[1 †], Pei Chen[1 †], Na Yang[1], Kazuyuki Aihara[2], Rui Liu[1]*, Luonan Chen[3,4,5,6]*

[1] School of Mathematics, South China University of Technology, Guangzhou 510640, China.

[2] International Research Center for Neurointelligence, The University of Tokyo Institutes for Advanced Study, The University of Tokyo, Tokyo 113-0033, Japan.

[3] Key Laboratory of Systems Health Science of Zhejiang Province, Hangzhou Institute for Advanced Study, University of Chinese Academy of Sciences, Chinese Academy of Sciences, Hangzhou 310024, China.

[4] Key Laboratory of Systems Biology, Shanghai Institute of Biochemistry and Cell Biology, Center for Excellence in Molecular Cell Science, Chinese Academy of Sciences, Shanghai 200031, China.

[5] Guangdong Institute of Intelligence Science and Technology, Hengqin, Zhuhai, Guangdong 519031, China.

[6] School of Life Science and Technology, ShanghaiTech University, Shanghai 201210, China.

† These authors contributed equally to this work and should be considered co-first authors.

* Correspondence: Luonan Chen, lnchen@sibcb.ac.cn; Rui Liu, scliurui@scut.edu.cn.

Contents

1. **Methods**......5

1.1 Delay embedding theorem for dynamical systems......5

1.2 Intrinsic workflow in the OCN Φ......6

1.3 Training the OCNS on a time-series dataset......7

2. **Supplementary Figures and Tables**......11

Figure S1. Summary of the reconstruction and prediction process for the original system according to the OCNS-based STI equations.......11

Figure S2. Sketch of the OCNS with 'sub-time' τ.......12

Figure S3. Illustration of the dynamical evolution of system $z\tau$ within the 'sub-time' τ. 13

Figure S4. Scheme employed by the OCNS for classification.......14

Figure S5. Forecasting the first three variables in a Lorenz system with a noise strength of $\sigma = 0.5$ using the OCNS.......15

Figure S6. Future state predictions for the other six variables in a Lorenz system under the noise-free situation.......16

Figure S7. Future state predictions for the other six variables in a Lorenz system with a noise strength of $\sigma = 0.5$.......17

Figure S8. Future state predictions for the other six variables in a Lorenz system with a noise strength of $\sigma = 1.0$.......18

Figure S9. Performance comparison based on Mean Absolute Error (MAE) among the OCNS and ten comparative methods on real-world datasets.......19

Figure S10. Visualization of the forecasting results for the oil temperature values in the $ETTh_1$ dataset.......20

Figure S11. Visualization of the forecasting results for the oil temperature values in the $ETTh_2$ dataset.......21

Figure S12. Visualization of the forecasting results for the oil temperature values in the $ETTm_1$ dataset.......22

Figure S13. Visualization of the forecasting results for the oil temperature values in the $ETTm_2$ dataset.......23

Figure S14. Visualization of the forecasting results for the air density values in the WTH_2 dataset.......24

Figure S15. Visualization of the forecasting results for the wet-bulb temperature values in the WTH_1 dataset.......25

Figure S16. Visualization of the forecasting results for the electricity consumption values in the ECL dataset.......26

Figure S17. Visualization of the forecasting results for the "index 3" values in the SMD dataset.......27

Figure S18. Schematic illustration of one step of the forward process in the OCN Φ. 28

Figure S19. Model efficiency comparison under (a) Lorenz, (b) WTH_2, (c) $ETTh_1$, and (d) $ETTh_2$ datasets. 30

Figure S20. Hyperparameter sensitivity of OCNS on $ETTh_1$ dataset with respect to the learning rate (a), the delay-embedding dimension S (b), the step size of the first delay K (c), the number of temporal delays D (d) in the OCN module, and four loss component weights $\lambda 1 - \lambda 4$ (e-h). 31

Figure S21. Convergence curves of the training loss for OCNS on the Lorenz (a), $ETTh_1$ (b), WTH_1 (c) and ECL (d) datasets. 32

Table S1. Results of an ablation study involving the exclusion of different loss terms from the fully supervised training loss, as defined in Eq. (S10). 33

Table S2. Prediction performance of OCNS across different data dimensions (n) for short-term high-dimensional systems. 34

Table S3. Classification performance comparison between generalized OCNS versions with and without the reconstruction module on five benchmark classification datasets. ...35

Table S4. Summary of the default parameters used by the OCNS for each forecasting dataset. 36

Table S5. Summary of the dataset statistics for the benchmark. 37

Table S6. Comparative overview of OCNS and time-delayed RC. 38

Table S7. Multivariate forecasting results obtained on the numerical Lorenz datasets and real-world datasets by the OCNS and other methods. 39

Table S8. Comparison of parameter requirements between the OCNS and ten other methods on real-world datasets, with comparable or superior OCNS performances as shown in Table S7. 40

Table S9. Multivariate forecasting results obtained on real-world datasets by the OCNS and other comparative methods with the same parameter scale. 41

Table S10. Performance comparisons conducted on classification tasks with respect to different time points N in the latent one-dimensional system. The text in bold denotes the best results obtained in each row. 42

3. Supplementary Sections 43

Section S1. Enhancements to the modules in the OCNS 43

Section S2. Dynamical systems and delay embedding theory 45

Section S3. Coupled Lorenz system 47

Section S4. Brief introduction to the real-world forecasting benchmark datasets 48

Section S5. Ablation study concerning the loss terms for forecasting 50

Section S6. Evaluating the OCNS on short-term time series across different data dimensions 50

Section S7. Generalization of time-series learning framework for categorization 50

Section S8. Data augmentation for the classification task 52

Section S9. Brief introduction to the comparative time-series forecasting algorithms in this study 52

Section S10. Model efficiency 55

Section S11. Hyperparameter robustness 55

Section S12. Convergence Analysis of OCNS 56

Section S13. Pseudocodes of OCNS 57

Section S14. Comparative Analysis of OCNS and time-delayed reservoir computing (RC) model 58

SI Reference 61

1. Methods

1.1 Delay embedding theorem for dynamical systems

The dynamics of a discrete-time dissipative system can be presented as

$$\boldsymbol{X}^{t+1} = \phi(\boldsymbol{X}^t) \tag{S1}$$

where $\phi: \mathbb{R}^n \to \mathbb{R}^n$ represents a nonlinear function, whose n-dimensional variables are denoted as vector $\boldsymbol{X}^t = [x_1^t, x_2^t, \ldots, x_n^t]'$ with the time superscript t and the vector transpose symbol " $'$ ". And such a system is evolving on a compact attractor $\mathcal{A} \subseteq \mathbb{R}^n$ with the Minkowski dimension/box-counting dimension $\text{boxdim}(\mathcal{A}) = d$. Actually, the delay embedding theory and its applications [1–4] provide the following facts.

For a C^2-diffeomorphism $\Psi: \mathcal{A} \to \mathcal{A}$ and a C^2-function $h: \mathcal{A} \to \mathbb{R}$, a generic property states that the mapping $\Upsilon_{\Psi,h}: \mathcal{A} \to \mathbb{R}^K$ is an embedding when integer $K > 2d$. That is,

$$\Upsilon_{\Psi,h}(X) = [h(X), h \circ \Psi(X), \ldots, h \circ \Psi^{K-1}(X)]' \tag{S2}$$

is generically an embedding where the " $\circ$ " symbol is the function composition operation [2]. In particular, letting $X = \boldsymbol{X}^t$, $\Psi = \phi$, $K = S$, and $h\big(\phi^{j-1}(\boldsymbol{X}^t)\big) = z^{t-S+j} \in \mathbb{R}$ $(j = 1,2, \ldots, S)$, where z^{t-S+j} denotes the states derived from a latent one-dimensional delay dynamical system z^t, the mapping above (Eq. (S2)) has the following form with $\Upsilon: \mathcal{A} \to \mathbb{R}^S$ and

$$\Upsilon(\boldsymbol{X}^t) = [z^{t-S+1}, \ldots, z^t]' = \boldsymbol{Z}^t. \tag{S3}$$

In this work, the OCN $\boldsymbol{\Phi}$ is designed to transform the spatial information $\boldsymbol{X}^t$ of the original system into time series $\boldsymbol{Z}^{t+i}$ $(i = 1,2, \ldots)$ of a latent variable in the form of $\boldsymbol{Z}^{t+i} = \boldsymbol{\Phi}(A\boldsymbol{X}^t, t+i) = \left[z^{t-S+1+i}, z^{t-S+2+i}, \ldots, z^{t+i}\right]' \in \mathbb{R}^S, i = 0,1,2 \ldots$, where A represents the input weight of the OCNS (Fig. 1a in the main text). Then, we can define a map as $\mathcal{O}^i\big[A, W_\varphi\big](\boldsymbol{X}^t) = \boldsymbol{Z}^{t+i}$, where W_φ indicates the parameters in the difference function $\varphi(\cdot)$ (see Eq. (1) of the main manuscript) of the one-dimensional delay dynamical system z^t. In other words, $\boldsymbol{Z}^t$ can be taken as a nonlinear observable of Eq.

(S1) after a transient phase, which can topologically reconstruct the attractor of the original dynamical system in Eq. (S1). Thus, with the above preparation and further delay embedding theory [1,2], we can immediately prove the following theorem.

Theorem 1. *Letting $S \geq 2d$ with d as the box-counting dimension of the attractor $\mathcal{A}$, then the map $O^i[A, W_\varphi] \in C^1(\mathcal{A}, \mathbb{R}^S)$ is generically an embedding.*

More details are given in Section S2. This theorem implies that $\boldsymbol{Z}^t$ (the time series of one variable) of the OCN can fully represent the state $\boldsymbol{X}^t$ (high-dimensional variables) of the original dynamical system and vice versa, i.e., OCN has the encoding ability and thus can be used for efficient deep learning.

Moreover, since the embedding is a one-to-one mapping, we can also derive its conjugate form $\Gamma: \mathbb{R}^S \rightarrow \mathbb{R}^n$ as $\boldsymbol{X}^t = \Upsilon^{-1}(\boldsymbol{Z}^t) = \Gamma(\boldsymbol{Z}^t)$. The above theory can be summarized as the following STI transformation equations:

$$\begin{cases} \Upsilon(\boldsymbol{X}^t) = \boldsymbol{Z}^t, \\ \boldsymbol{X}^t = \Gamma(\boldsymbol{Z}^t), \end{cases} \tag{S4}$$

where $\Upsilon: \mathbb{R}^n \rightarrow \mathbb{R}^S$ and $\Gamma: \mathbb{R}^S \rightarrow \mathbb{R}^n$ are nonlinear differentiable functions satisfying $\Upsilon \circ \Gamma = id$, the "∘" symbol represents the function composition operation and id denotes the identity function. Clearly, based on Eq. (S4) the time series $\boldsymbol{Z}^t$ of the single variable z can represent the high-dimensional spatial data $\boldsymbol{X}^t$, in contrast to existing deep learning framework representing such spatial data by one vector, thus opening a new way for deep learning with one neuron.

1.2 Intrinsic workflow in the OCN $\boldsymbol{\Phi}$

Given a high-dimensional spatial vector $\boldsymbol{X}^t = [x_1^t, x_2^t, \dots x_n^t]' \in \mathbb{R}^n$ at time t, the OCN $\boldsymbol{\Phi}$ construct the delayed vectors $\boldsymbol{Z}^{t+i} = \boldsymbol{\Phi}(A\boldsymbol{X}^t, t+i) = [z^{t-S+1+i}, z^{t-S+2+i}, \dots, z^{t+i}]' \in \mathbb{R}^S$ for the current and future time points $t+i, i = 0,1,2,\dots,l$ from the latent one-dimensional delay dynamical system z^t, where l denotes the prediction horizon of the OCNS, S is the delay-embedding dimension. That is to say, the delay vector $\boldsymbol{Z}^{t+i}$ is entirely derived from the states of the one-

dimensional delay dynamical system z^t. As the system state of z^t automatically evolves in discrete time according to the delay difference function $\varphi(\cdot)$ (see Eq. (1) of the main manuscript), the OCN $\mathbf{\Phi}$ can be conceptualized as a vector-valued function with the following form:

$$\mathbf{\Phi}(A\mathbf{X}^t, t+i) = \begin{bmatrix} \varphi\big(z(t-S+1+i-K), \dots, z(t-S+1+i-(K+D-1)); t-S+1+i, W_\varphi\big) \\ \varphi\big(z(t-S+2+i-K), \dots, z(t-S+2+i-(K+D-1)); t-S+2+i, W_\varphi\big) \\ \dots \\ \varphi\big(z(t+i-K), \dots, z(t+i-(K+D-1)); t+i, W_\varphi\big) \end{bmatrix} = \mathbf{Z}^{t+i}, \quad \text{(S5)}$$

where $i = 0,1,2, \dots, l$. In other words, during training ($i = 0$), the OCN $\mathbf{\Phi}$ behaves in the form of $\mathbf{\Phi}(A\mathbf{X}^t, t)$ and yields a sequence $\mathbf{Z}^t$ with a length of S. Each element in $\mathbf{Z}^t$ signifies the result of applying $\varphi(\cdot)$, representing the state of the delay system z^t at the corresponding time stamp. For prediction ($i \geq 1$), given that the dynamics of the original high-dimensional system have already been captured by the OCN $\mathbf{\Phi}$, future information can be forecasted through iterative forward propagation based on the existing latent sequence. Due to the use of the delay-embedding scheme, there exist $S-1$ shared latent temporal states between two temporally adjacent delayed vectors. In essence, this process involves advancing the latent delayed system z^t by one step forward. Consequently, the outcome of one-step forward temporal process in the OCN $\mathbf{\Phi}$, i.e., from $\mathbf{Z}^t = \mathbf{\Phi}(A\mathbf{X}^t, t)$ to $\mathbf{Z}^{t+1} = \mathbf{\Phi}(A\mathbf{X}^t, t+1)$, is attained by applying the function $\varphi(\cdot)$ to the existing system states of z^t for the next one step. In this manner, through iteratively applying the function $\varphi(\cdot)$ to the system z^t, multistep-ahead delayed vectors $\mathbf{Z}^{t+i}, i = 1,2, \dots, l$ containing information about the future states ahead of t can be obtained, even extending the prediction horizon to infinity.

1.3 Training the OCNS on a time-series dataset

For multivariate time-series forecasting tasks, the core of the OCNS is to determine the appropriate OCN $\mathbf{\Phi}$, input weight A and output weight B. Through integrating the delay embedding theorem by incorporating a dynamical manifold regularization into the loss function, the training procedure of the OCNS is supervised by a combined loss, including a reconstruction loss $\mathcal{L}_{\mathcal{R}}$, a prediction loss $\mathcal{L}_{\mathcal{P}}$, an embedding consistency loss $\mathcal{L}_{\mathcal{EC}}$, and an embedding divergence loss $\mathcal{L}_{\mathcal{ED}}$. $\mathcal{L}_{\mathcal{EC}}$ represents the dynamical manifold regularization because this regularization term enforces the output of the OCN

to be confined within a specific manifold (the delay embedding space), thereby capturing the underlying structure of the original system.

Reconstruction loss. $\mathcal{L}_{\mathcal{R}} = \frac{1}{m}\sum_{i=1}^{m}\left\|\boldsymbol{X}^{t+i} - \widehat{\boldsymbol{X}}^{t+i}\right\|_2^2$, where $\boldsymbol{X}^{t+i}$ and $\widehat{\boldsymbol{X}}^{t+i}$ are the ground truth and reconstructed high-dimensional input time series ($i = 1, \dots, m$), respectively. In the OCNS, we reconstruct the estimated known states $\boldsymbol{X}^t$ from $\boldsymbol{Z}^t$ through the output weight B, i.e., the conjugate equation of Eq. (3) of the main manuscript.

Prediction loss. $\mathcal{L}_{\mathcal{P}} = \frac{1}{l}\sum_{i=1}^{l}\left\|\boldsymbol{X}^{t+m+i} - \widehat{\boldsymbol{X}}^{t+m+i}\right\|_2^2$, where $\boldsymbol{X}^{t+m+i}$ and $\widehat{\boldsymbol{X}}^{t+m+i}$ denote the ground truth and estimated high-dimensional future time series ($i = 1,2, \dots, l$), respectively.

Embedding consistency loss (dynamical manifold regularization). For an input time series $[\boldsymbol{X}^{t+1}, \boldsymbol{X}^{t+2}, \dots, \boldsymbol{X}^{t+m}]$ with a length of m, the OCNS transforms the spatial information at each time point into the corresponding estimated delay vectors $\{\widehat{\boldsymbol{Z}}^{t+1}, \widehat{\boldsymbol{Z}}^{t+2}, \dots, \widehat{\boldsymbol{Z}}^{t+m}\}$ containing the temporal information of the latent delay dynamical system z^t, while the estimated delay vectors $\{\widehat{\boldsymbol{Z}}^{t+m+1}, \widehat{\boldsymbol{Z}}^{t+m+2}, \dots, \widehat{\boldsymbol{Z}}^{t+m+l}\}$, which contain the future latent temporal information corresponding to the time series to be predicted $[\boldsymbol{X}^{t+m+1}, \boldsymbol{X}^{t+m+2}, \dots, \boldsymbol{X}^{t+m+l}]$, can be directly derived from the determined latent delay dynamical system z^t. Corresponding to the delay-embedding matrices in Eq. (4) and Eq. (5) of the main manuscript, all the estimated delay vectors can be merged into one delay-embedding matrix as follows:

$$\left[\widehat{\boldsymbol{Z}}^{t+1}, \dots, \widehat{\boldsymbol{Z}}^{t+m}, \widehat{\boldsymbol{Z}}^{t+m+1}, \dots, \widehat{\boldsymbol{Z}}^{t+m+l}\right]$$
$$= \begin{bmatrix} \hat{z}_{t+1}^{t-S+2} & \cdots & \hat{z}_{t+m}^{t-S+m+1} & \hat{z}_{t+m+1}^{t-S+m+2} & \cdots & \hat{z}_{t+m+l}^{t-S+m+l+1} \\ \hat{z}_{t+1}^{t-S+3} & \cdots & \hat{z}_{t+m}^{t-S+m+2} & \hat{z}_{t+m+1}^{t-S+m+3} & \cdots & \hat{z}_{t+m+l}^{t-S+m+l+2} \\ \vdots & \ddots & \vdots & \vdots & \ddots & \vdots \\ \hat{z}_{t+1}^{t+1} & \cdots & \hat{z}_{t+m}^{t+m} & \hat{z}_{t+m+1}^{t+m+1} & \cdots & \hat{z}_{t+m+l}^{t+m+l} \end{bmatrix}_{S\times(m+l)}, \quad \text{(S6)}$$

where $\hat{z}_{t+i}^{t-S+i+j}$ ($i = 1,2, \dots, m + l; j = 1,2, \dots, S$) denotes the j-th estimated latent temporal state for $\boldsymbol{X}^{t+i}$. Regarding the estimated latent temporal state at the j-th row and i-th column in this matrix (Eq. (S6)) as $\tilde{z}_{j,i} = \hat{z}_{t+i}^{t-S+i+j}$, a temporally self-constrained condition with $i > j$:

$$\tilde{z}_{j,i} = \tilde{z}_{j+1,i-1} \tag{S7}$$

should be satisfied according to the delay-embedding scheme. Thus, the embedding consistency loss can be computed as:

$$\mathcal{L}_{\mathcal{EC}} = \frac{1}{(m+l) \times S} \sum_{j=1}^{S} \sum_{i=1}^{m+l} \left(\tilde{z}_{j,i} - \bar{z}^{t-S+i+j}\right)^2, \tag{S8}$$

where $\bar{z}^{t-S+i+j}$ denotes the mean state calculated from all estimated latent states corresponding to time point $t-S+i+j$ with $i = 1,2,\dots,m+l; j = 1,2,\dots,S$. Clearly, $\mathcal{L}_{\mathcal{EC}}$ is constructed from the temporally self-constrained conditions in Eq. (S7). An intuitive understanding of the dynamical manifold regularization is that it enforces the latent delay states $\tilde{z}_{j,i}$ corresponding to the same time point $t-S+i+j$ to be identical, thereby preserving the temporal consistency of the delay-embedding matrix (Eq. (S6)) during the training procedure. In other words, the embedding consistency loss $\mathcal{L}_{\mathcal{EC}}$ imposes a strong dynamical constraint on the latent one-dimensional delay dynamical system z^t to fulfill the delay-embedding scheme.

Embedding divergence loss. Given the delay-embedding matrix (Eq. (S6)), we can obtain the aggregated latent temporal states $\mathcal{Z}^t = \left\{\bar{z}^{t-S+i}\right\}_{i=1}^{m+l+S}$, where $\bar{z}^{t-S+i}$ denotes the mean state, calculated from all estimated latent states corresponding to time point $t-S+i$ with $i = 1,2,\dots,m+l+S$ (as shown in Eq. (S8)). Subsequently, the embedding divergence loss is computed as:

$$\mathcal{L}_{\mathcal{ED}} = \frac{1}{\left(\mathrm{SD}(\mathcal{Z}^t)\right)^2}, \tag{S9}$$

where $\mathrm{SD}(\mathcal{Z}^t)$ is the standard deviation of all the latent temporal states. The embedding divergence loss guarantees a large variance among the states in the latent one-dimensional delay dynamical system z^t, so that it can mitigate the risk of trivial solutions emerging and z^t can better represent the dynamics of the original system.

In our implementation, the OCNS is trained by minimizing the weighted combined loss:

$$\mathcal{L}_{\mathcal{F}ull} = \lambda_1 \mathcal{L}_{\mathcal{R}} + \lambda_2 \mathcal{L}_{\mathcal{P}} + \lambda_3 \mathcal{L}_{\mathcal{EC}} + \lambda_4 \mathcal{L}_{\mathcal{ED}}, \tag{S10}$$

where the hyperparameters $\lambda_1, \lambda_2, \lambda_3, \lambda_4$ control the contributions of different loss

terms during the training process. We have analyzed the convergence properties of OCNS, and the relevant details are provided in Section S12. To further explore the impacts of the different losses used in $\mathcal{L}_{\mathcal{F}u\ell\ell}$ (Eq. (S10)) on the performance of the time-series forecasting task, we refer to Section S5 for the related ablation study. The weight parameters in the loss function and the other standard parameters used by the OCNS for all validation datasets are summarized in Table S4. A detailed analysis of OCNS's hyperparameter robustness is provided in Section S11.

2. Supplementary Figures and Tables

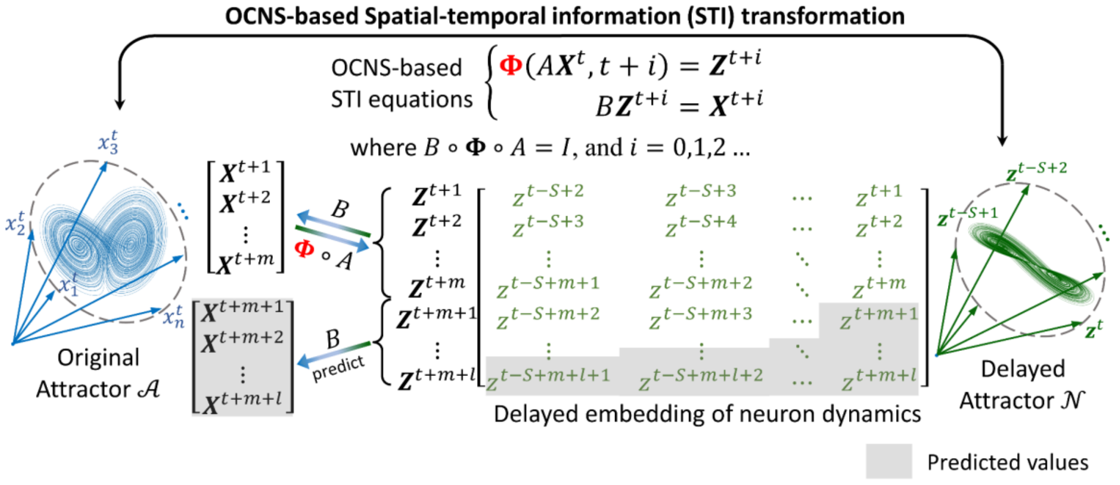

Figure S1. Summary of the reconstruction and prediction process for the original system according to the OCNS-based STI equations. Leveraging the theoretical concept of time-delay dynamical systems [5–7] and the delay embedding theorem [1,2,8], the OCNS-based STI equations, comprising both primary (the first formula) and conjugate (the second one) STI equations, are introduced. Given a known time series with a length of m, the OCNS is designed to forecast the next l steps in a multivariate manner using the OCNS-based STI equations. First, relying on the conjugate OCNS-based STI equation, the OCNS reconstructs/decodes the m known spatial vectors $[\boldsymbol{X}^{t+1}, \boldsymbol{X}^{t+2}, \ldots, \boldsymbol{X}^{t+m}]$ from the corresponding delayed/temporal vectors $[\boldsymbol{Z}^{t+1}, \boldsymbol{Z}^{t+2}, \ldots, \boldsymbol{Z}^{t+m}]$, while the primary equation (with the OCN $\boldsymbol{\Phi}$ and the input weight A) transforms the spatial vectors into delayed/temporally vectors. During the prediction phase, we iteratively advance the states of the latent one-dimensional delay dynamical system (denoted as z^t), which captures the inherent dynamics that are presented in the original system, to obtain the subsequent l latent temporal states $z^{t+m+1}, z^{t+m+2}, \ldots, z^{t+m+l}$ containing the future information/dynamics of the original system. Subsequently, we can construct a delay-embedding matrix by combining the known and predicted subvectors, further forecasting the next l spatial vectors $[\boldsymbol{X}^{t+m+1}, \boldsymbol{X}^{t+m+2}, \ldots, \boldsymbol{X}^{t+m+l}]$ through the output weight B.

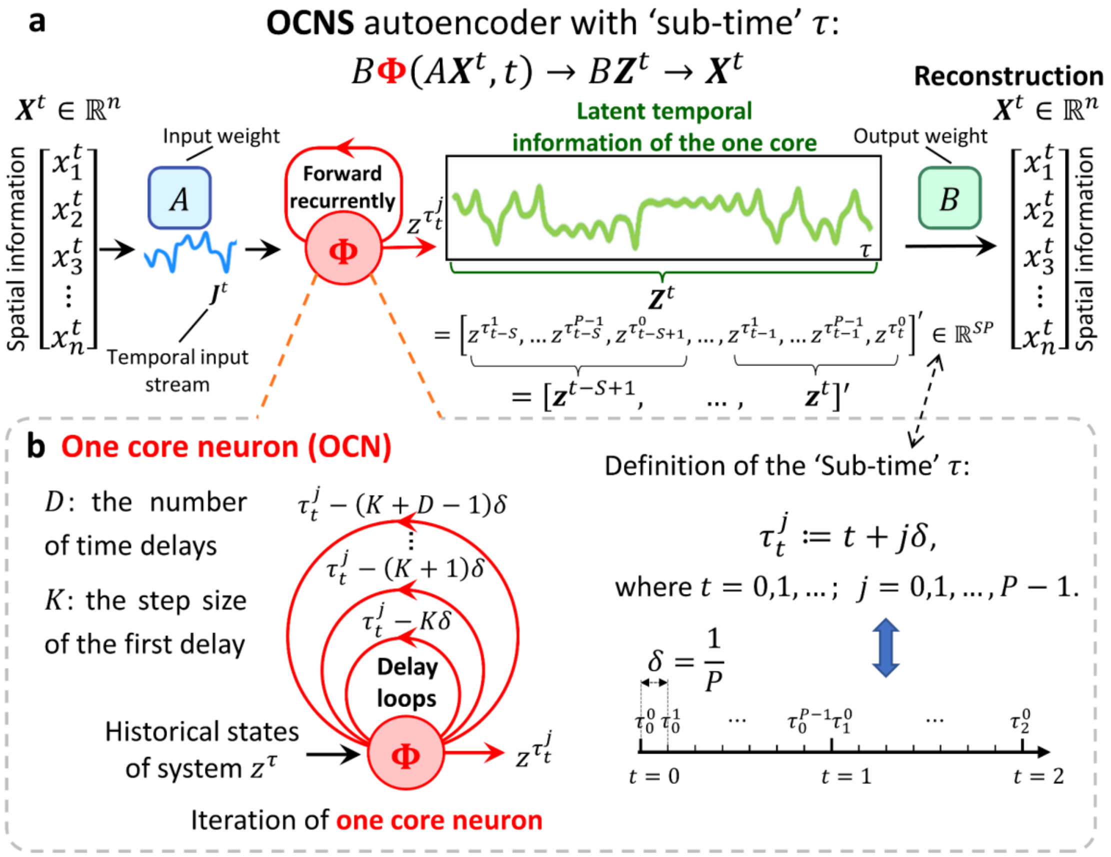

Figure S2. Sketch of the OCNS with 'sub-time' τ**. (a)** The OCNS framework incorporating 'sub-time' τ is an extension of the vanilla OCNS outlined in the main text. This integration of 'sub-time' into the OCN $\mathbf{\Phi}$ is achieved through time multiplexing. **(b)** The OCN $\mathbf{\Phi}$ processes the inner delayed dynamics of the latent one-dimensional system z^τ in the latent time τ with interval $\delta = 1/P$ and $P > 2$.

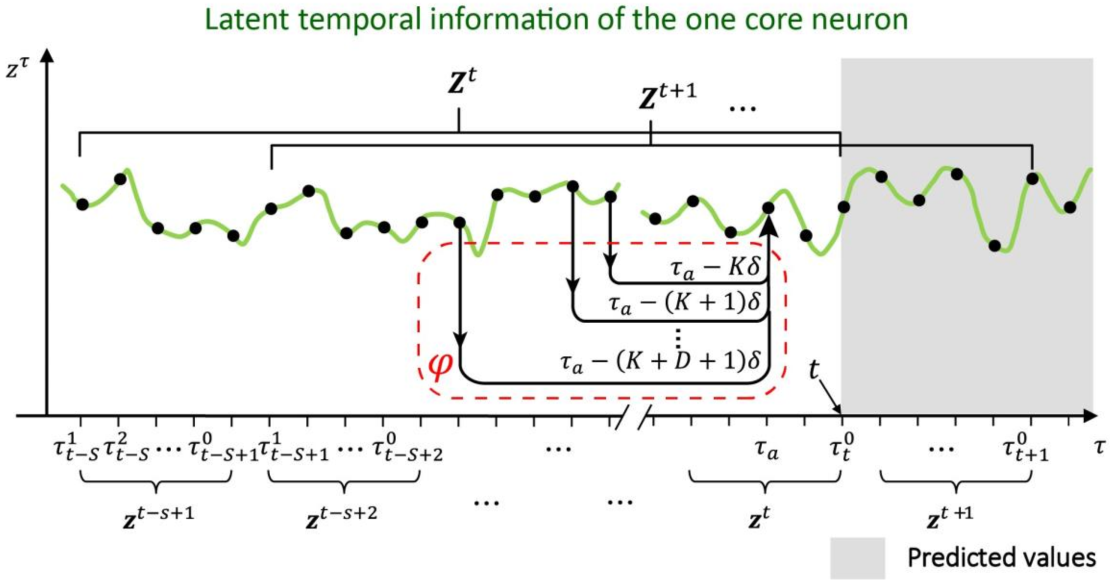

Figure S3. Illustration of the dynamical evolution of system z^τ within the 'sub-time' τ. Each of the temporal grid points is denoted as $\tau_t^j = t + j\delta$ with $j = 0,1,\dots,P-1$. Dynamic evolution of the latent one-dimensional delay dynamical system z^τ within the OCN $\boldsymbol{\Phi}$ is governed by a difference function $\varphi(\cdot)$ (Eq. (S12)). Due to the delay loops, the system state z^{τ_a} at a certain time point τ_a is considered by the delay feedback signals $\{z^{\tau_a-K\delta}, z^{\tau_a-(K+1)\delta}, \dots, z^{\tau_a-(K+D-1)\delta}\}$ with D time delays $\{K\delta, (K+1)\delta, \dots, (K+D-1)\delta\}$. Generally, the constant K is fixed, which implies that the advancement exhibited by the evolving delay system is K steps during each iteration. Based on this latent one-dimensional delay dynamical system z^τ, a delayed/temporally partitioned vector $\boldsymbol{Z}^t = [\boldsymbol{z}^{t-S+1}, \boldsymbol{z}^{t-S+2}, \dots, \boldsymbol{z}^t]' \in \mathbb{R}^{SP}$ is constructed, where each row vector $\boldsymbol{z}^{t-S+k} = \left[z^{\tau^1_{t-S+k-1}}, z^{\tau^2_{t-S+k-1}}, \dots, z^{\tau^{P-1}_{t-S+k-1}}, z^{\tau^0_{t-S+k}}\right] \in \mathbb{R}^P$, $k = 1,2,\dots,S$ is a latent temporal subvector, S is the delay-embedding dimensionality, and " ' " is the transpose of a vector.

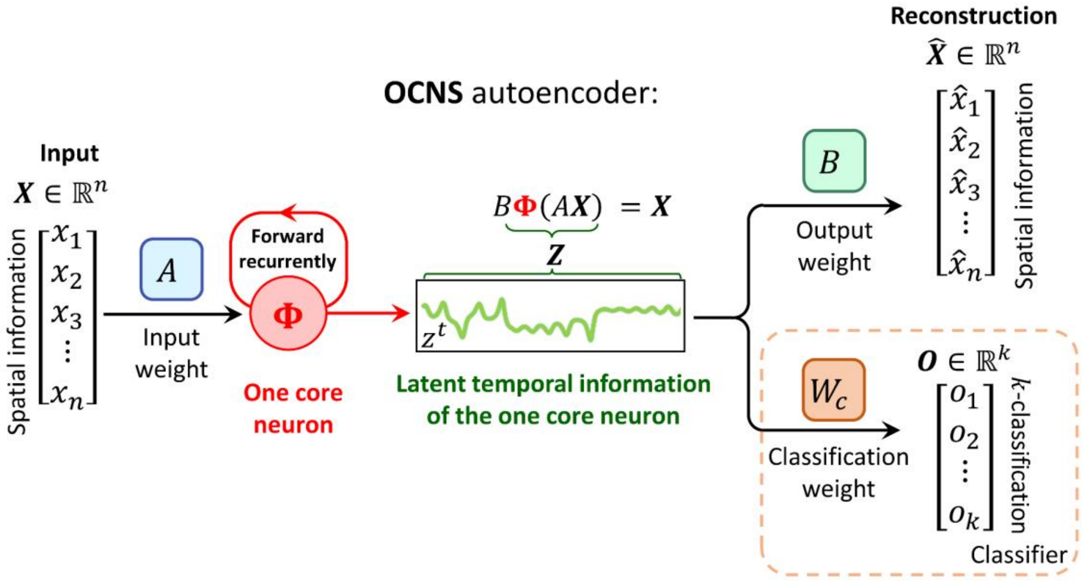

Figure S4. Scheme employed by the OCNS for classification. The generalized framework for using the OCNS is established by integrating a k-classification classifier into the original OCNS autoencoder. Specifically, the entire pipeline is a multi-task learning procedure, signifying that the latent dynamics or time series $\boldsymbol{Z}$ learned from the input spatial vector are simultaneously used for classification and reconstruction.

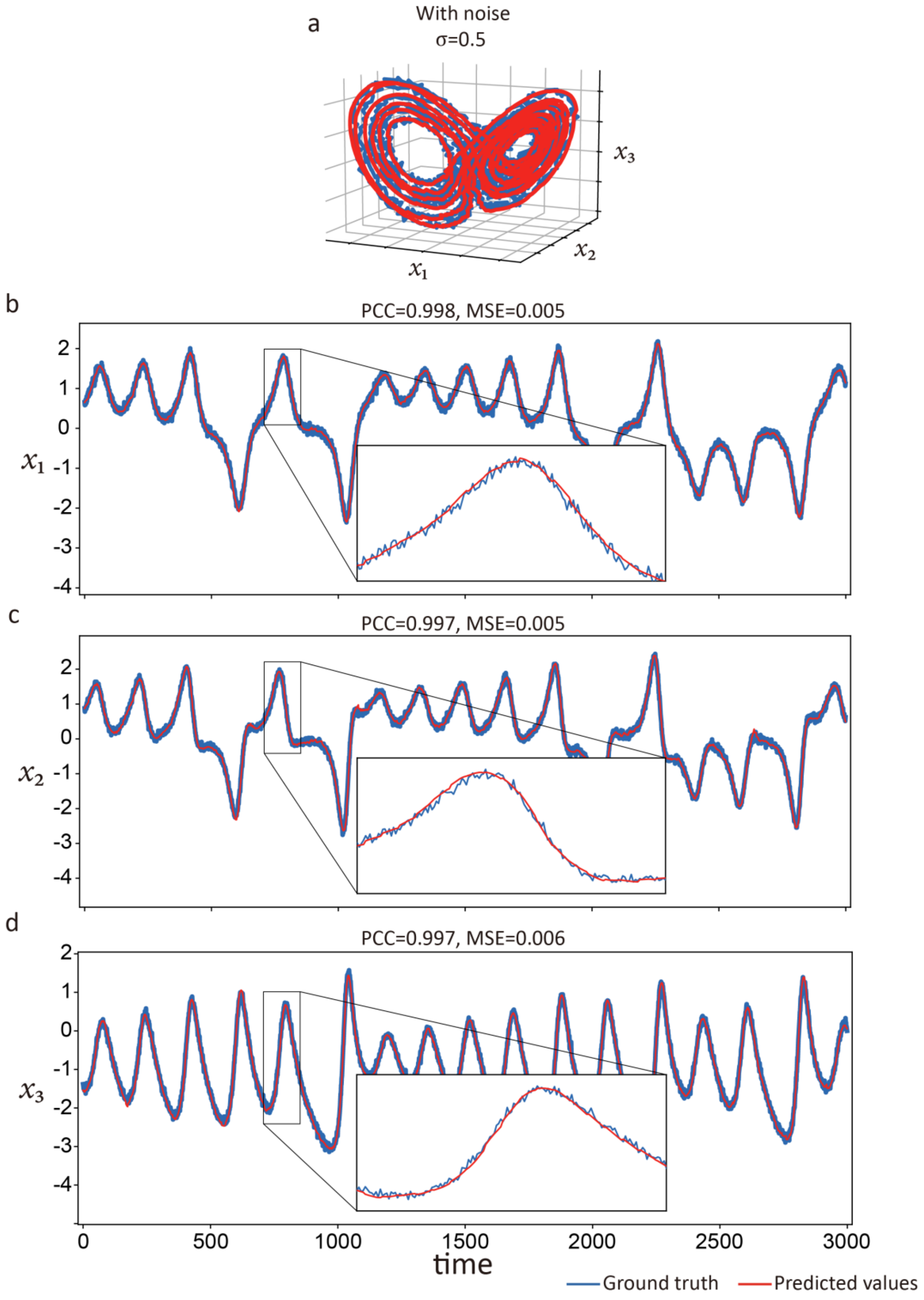

Figure S5. Forecasting the first three variables in a Lorenz system with a noise strength of $\sigma = 0.5$ using the OCNS. (a) Ground truth (blue) and predicted (red) 3D attractors. **(b-d)** The separate results are the predictions obtained for the first three variables x_1, x_2, x_3, which correspond to the 3D attractor in **(a)**.

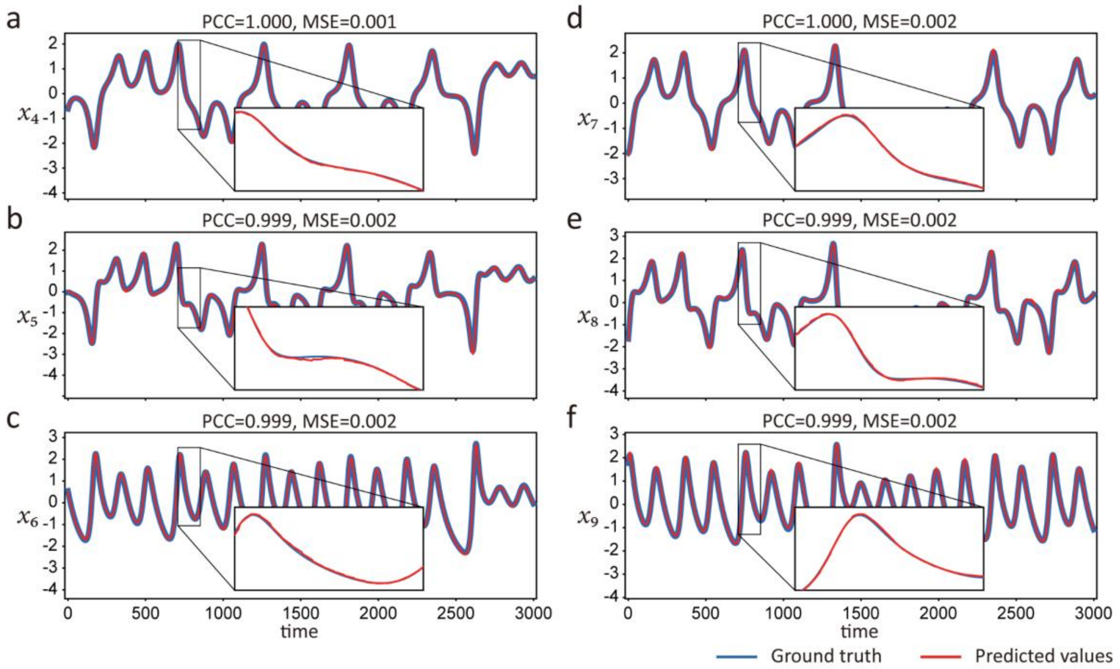

Figure S6. Future state predictions for the other six variables in a Lorenz system under the noise-free situation. The long-term predicted values overlay the ground-truth data for $x_4, x_5, x_6, x_7, x_8, x_9$, as shown in **(a-f)**.

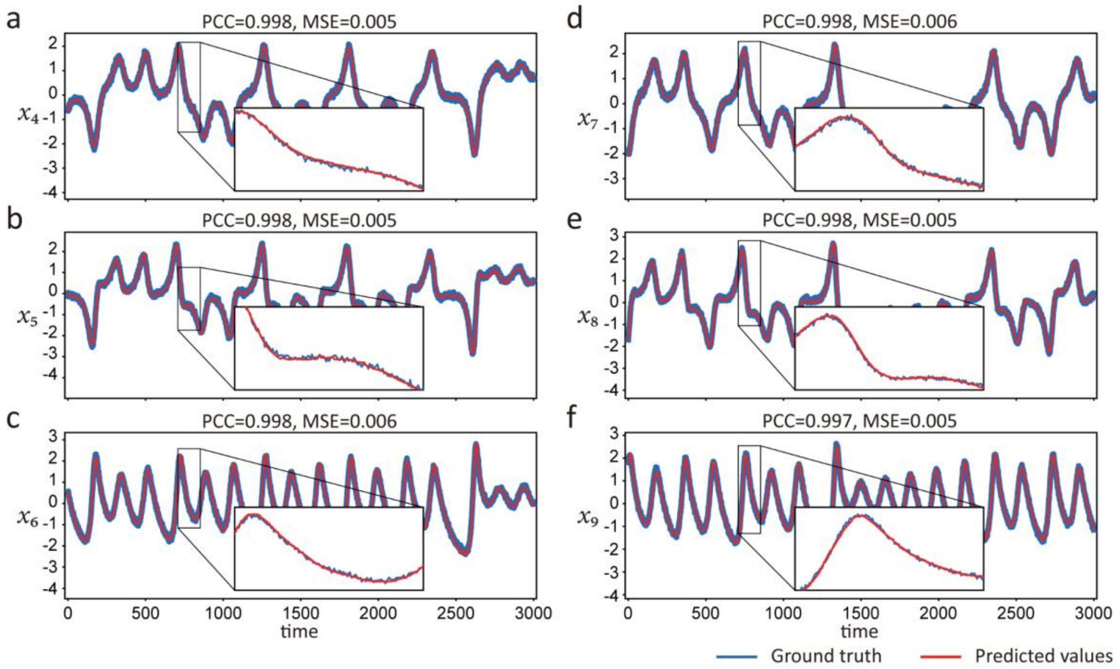

Figure S7. Future state predictions for the other six variables in a Lorenz system with a noise strength of $\sigma = 0.5$. The long-term forecasting results produced for $x_4, x_5, x_6, x_7, x_8, x_9$ are shown in **(a-f)**.

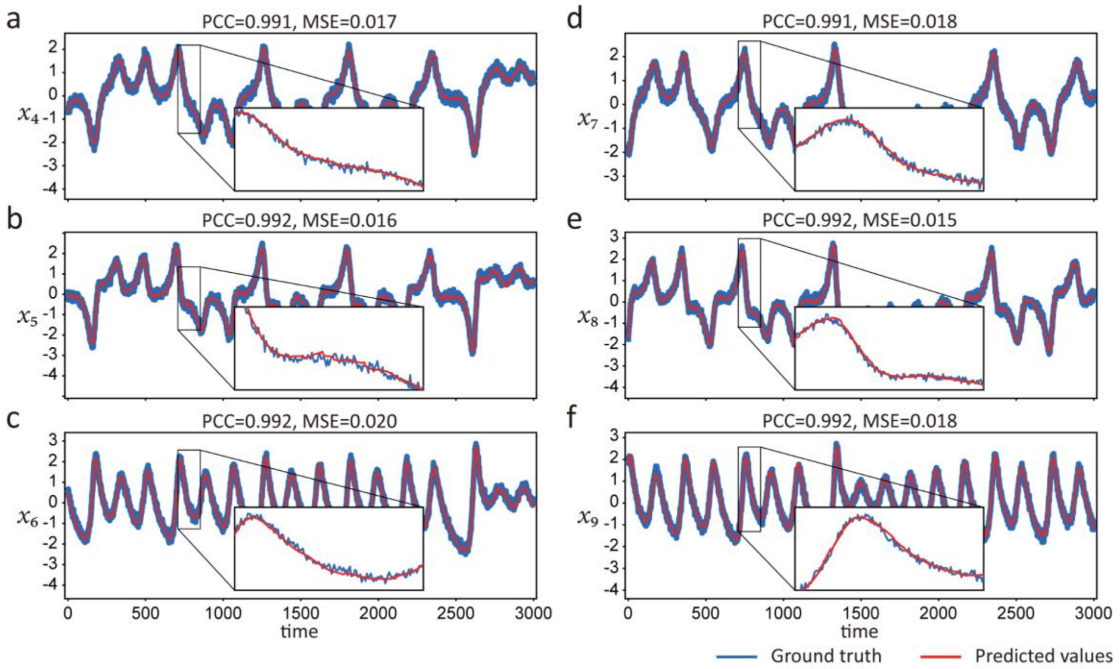

Figure S8. Future state predictions for the other six variables in a Lorenz system with a noise strength of $\boldsymbol{\sigma = 1.0}$. The long-term forecasting results produced for $x_4, x_5, x_6, x_7, x_8, x_9$ are shown in **(a-f)**.

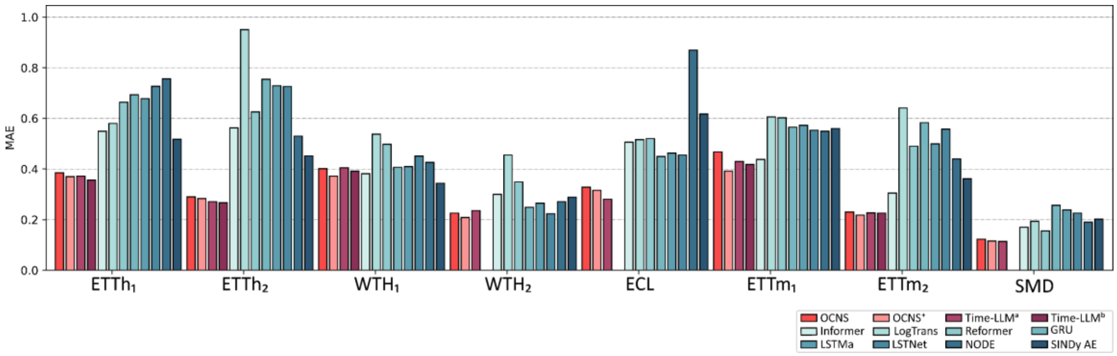

Figure S9. Performance comparison based on Mean Absolute Error (MAE) among the OCNS and ten comparative methods on real-world datasets. The MAEs of Time-LLM[b] on WTH_2, ECL and SMD datasets could not be obtained because the training time exceeded the 24-hour time limit.

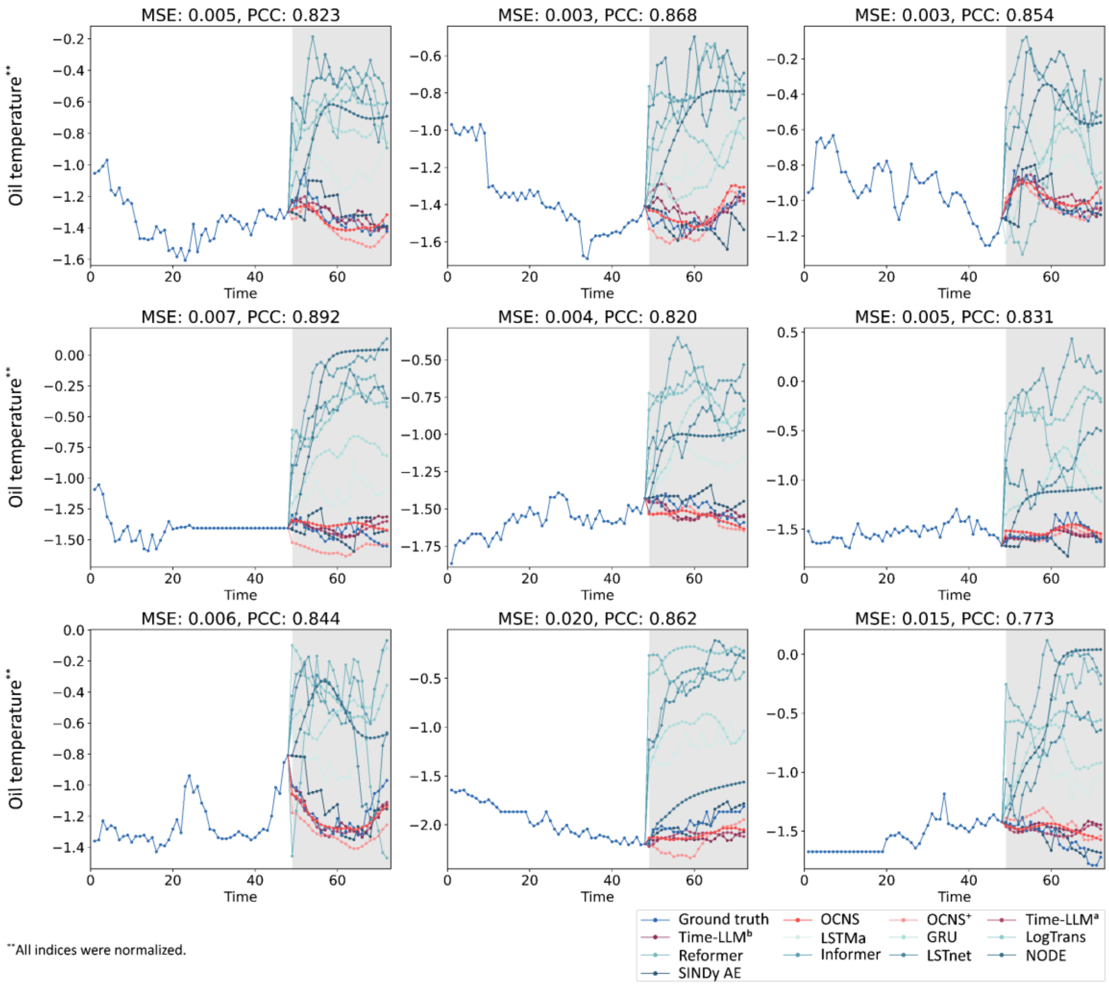

Figure S10. Visualization of the forecasting results for the oil temperature values in the ETTh$_1$ dataset.

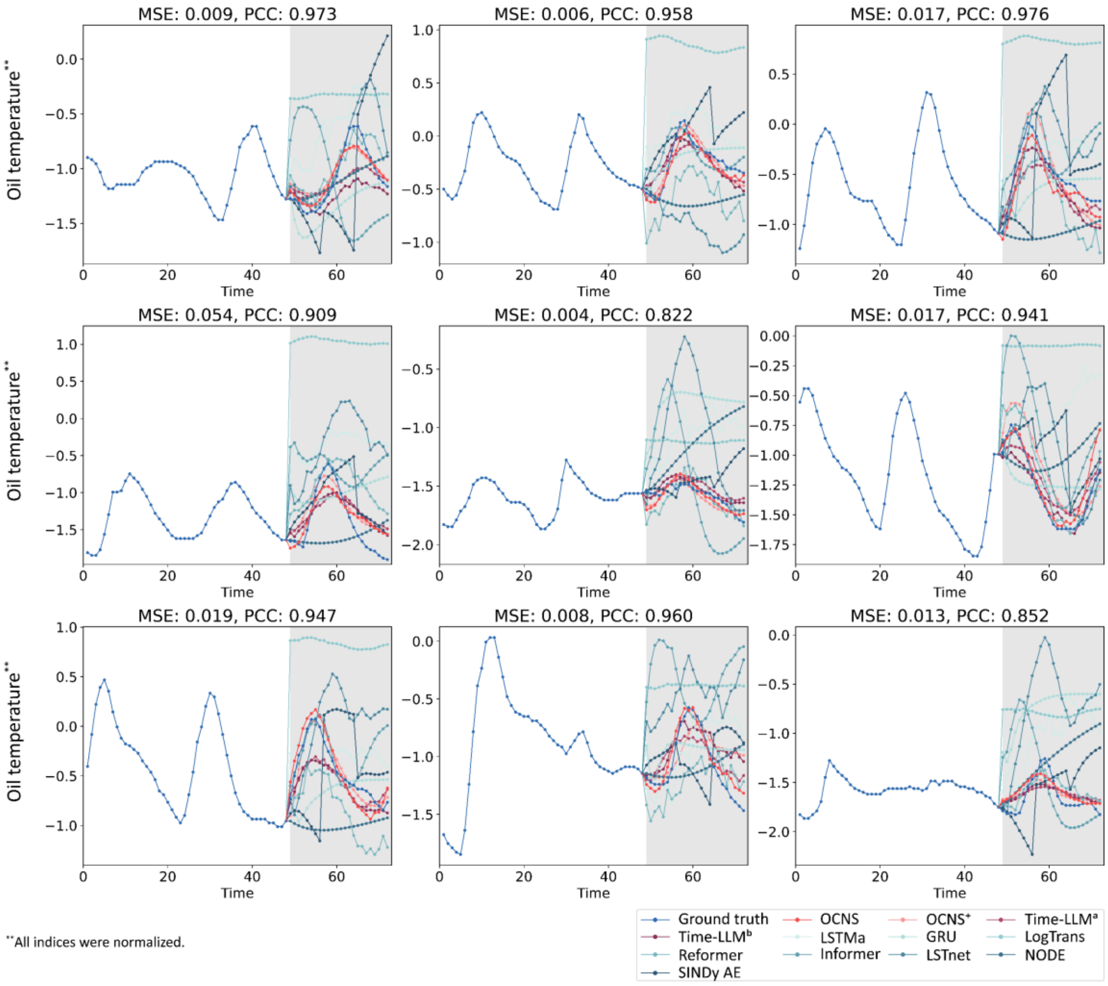

Figure S11. Visualization of the forecasting results for the oil temperature values in the ETTh2 dataset.

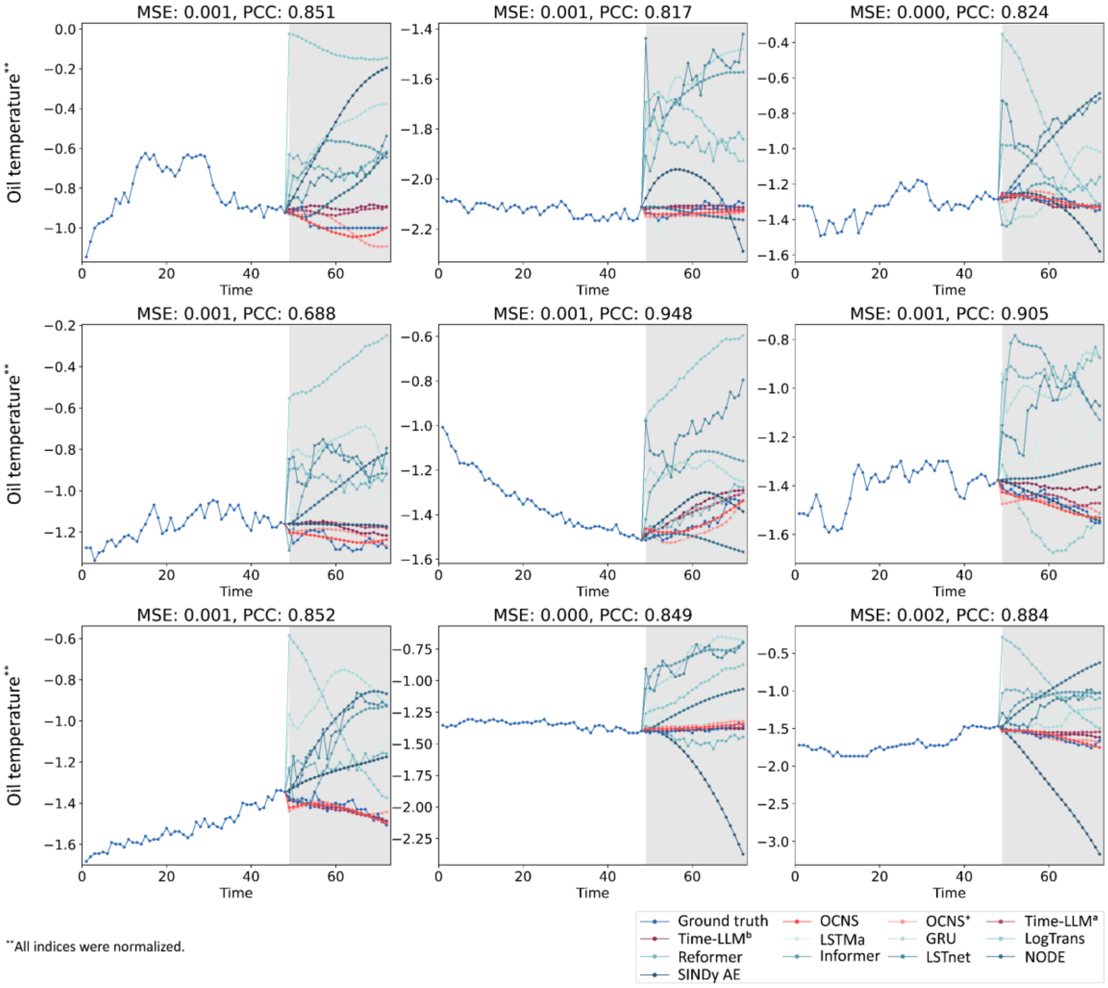

Figure S12. Visualization of the forecasting results for the oil temperature values in the ETTm1 dataset.

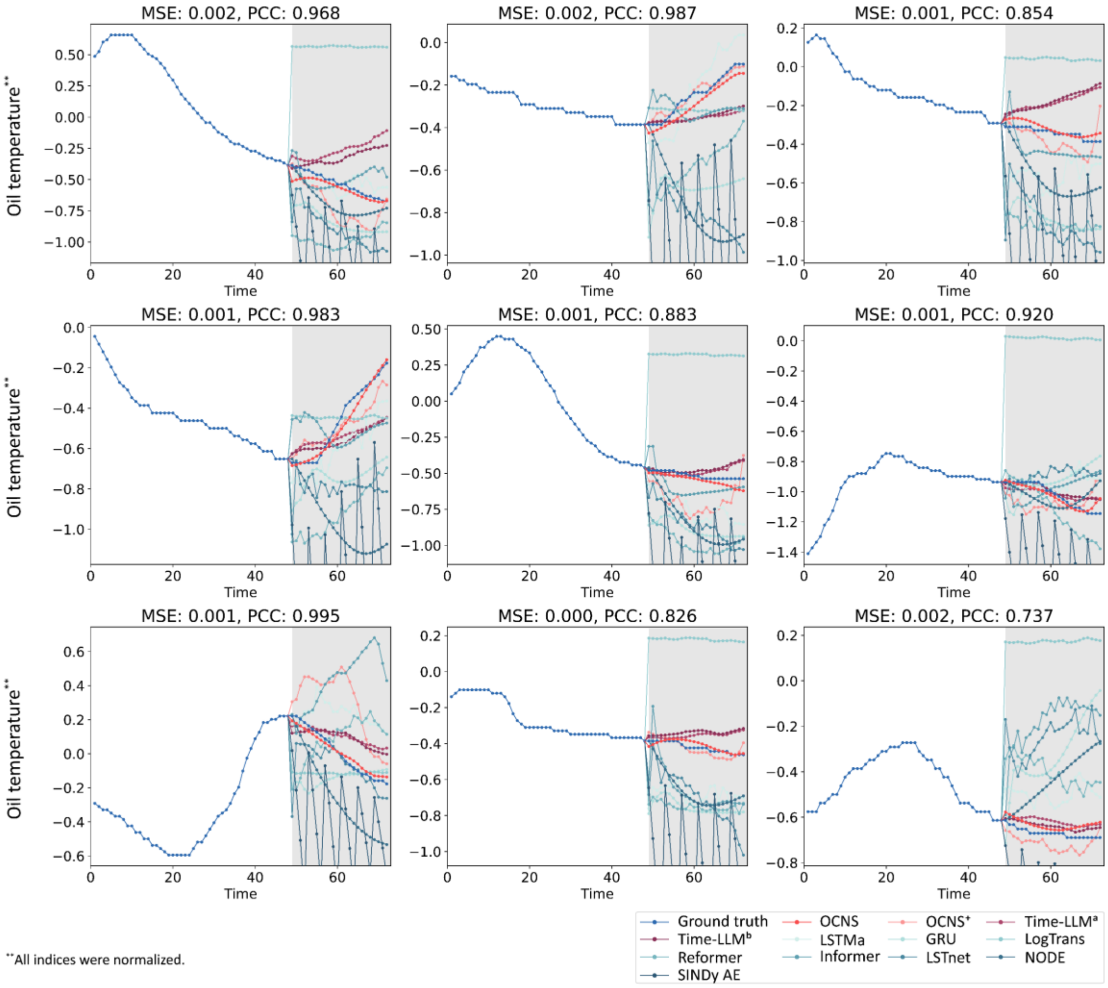

Figure S13. Visualization of the forecasting results for the oil temperature values in the ETTm2 dataset.

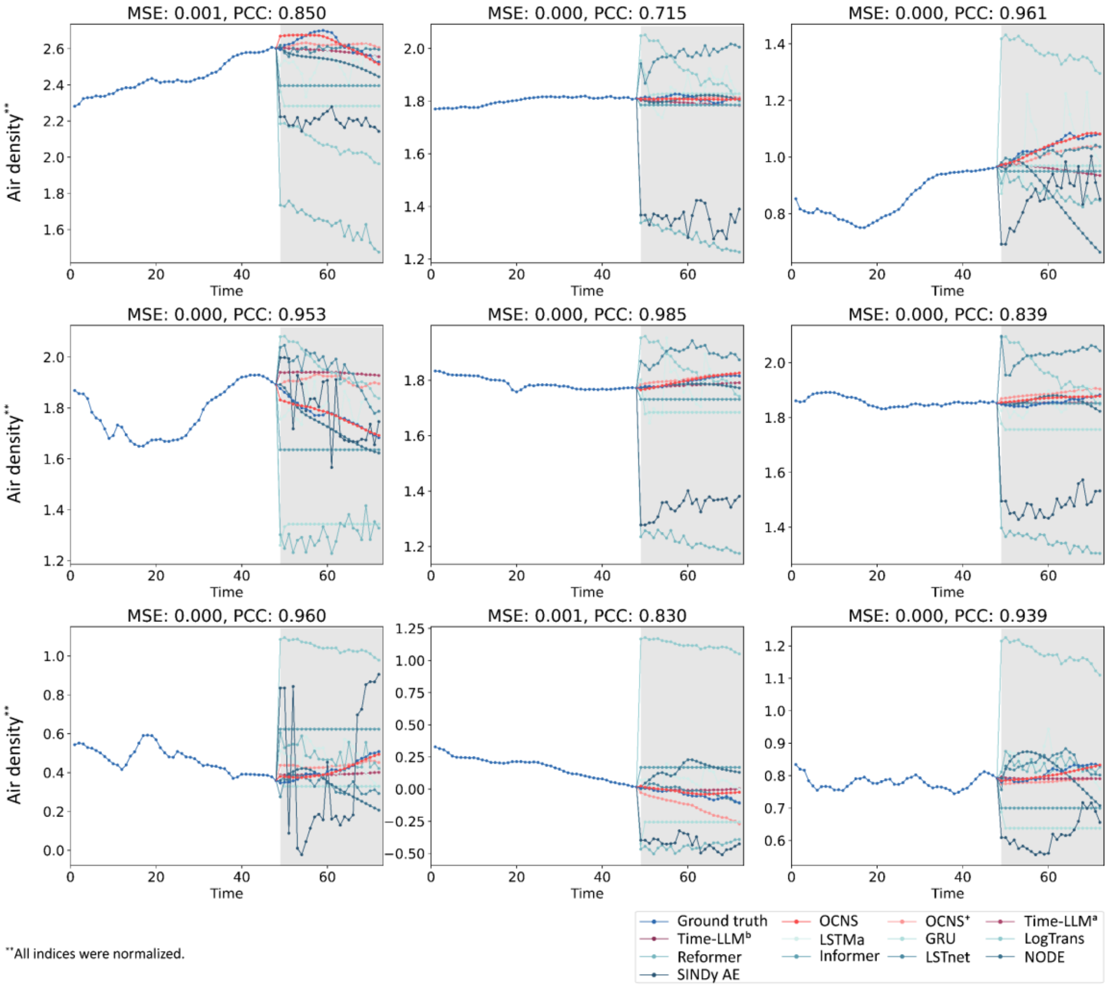

Figure S14. Visualization of the forecasting results for the air density values in the WTH_2 dataset.

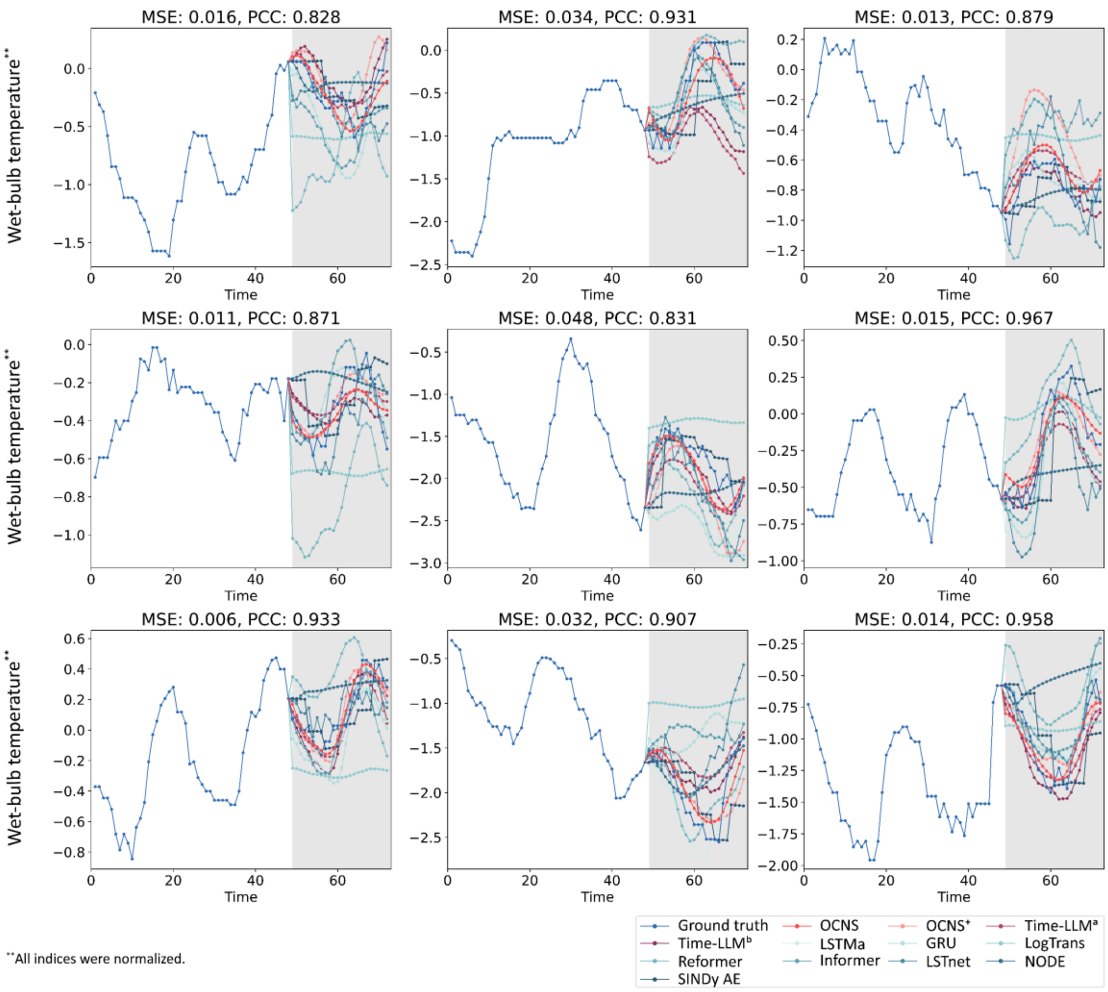

Figure S15. Visualization of the forecasting results for the wet-bulb temperature values in the WTH$_1$ dataset.

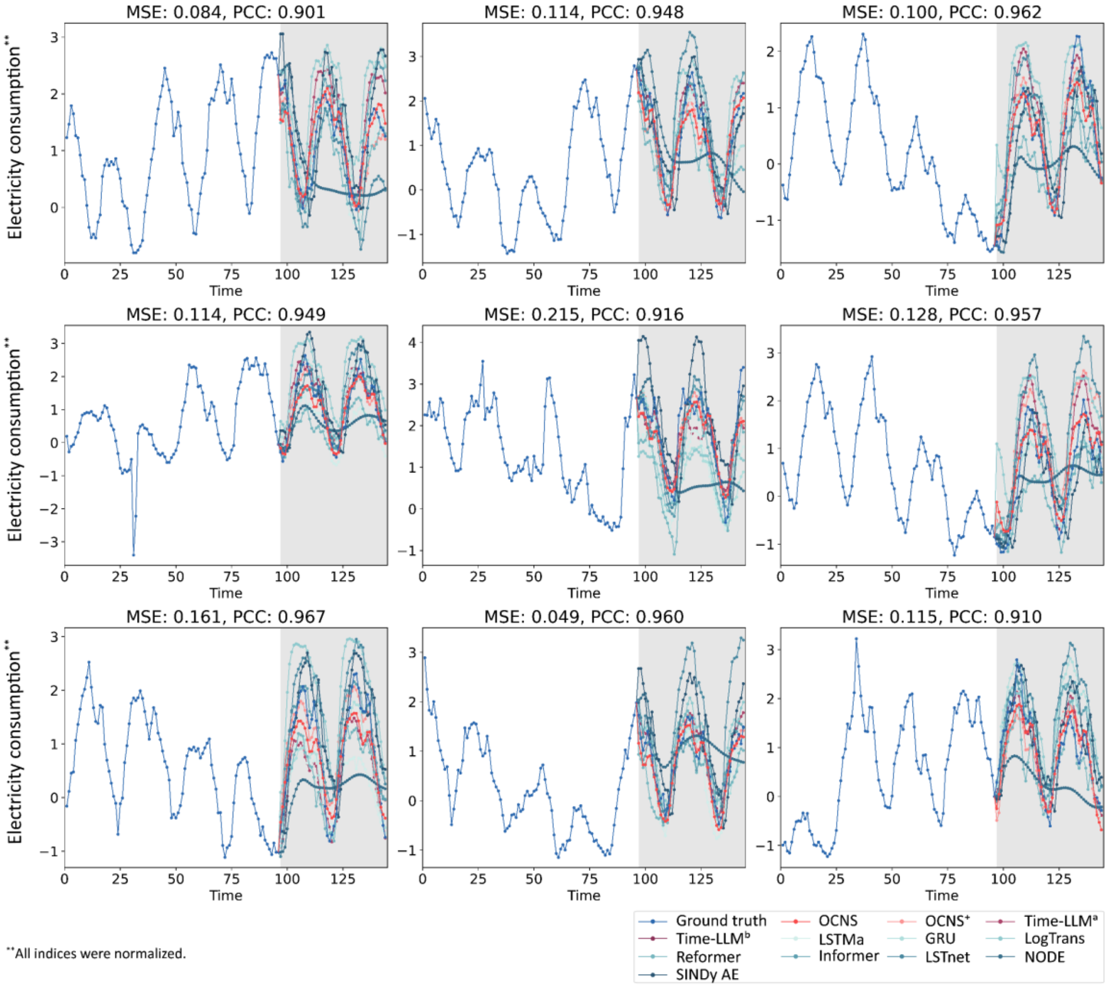

Figure S16. Visualization of the forecasting results for the electricity consumption values in the ECL dataset.

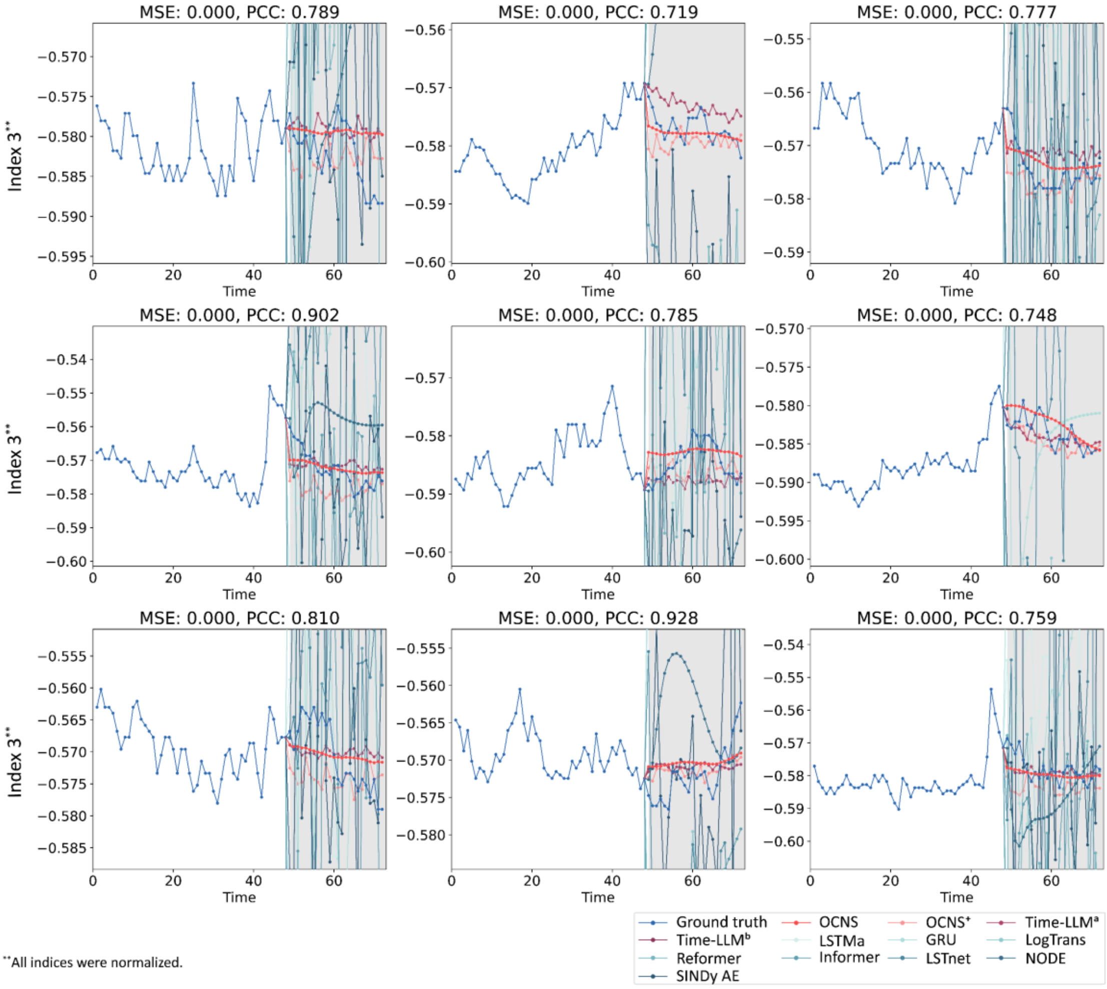

Figure S17. Visualization of the forecasting results for the "index 3" values in the SMD dataset.

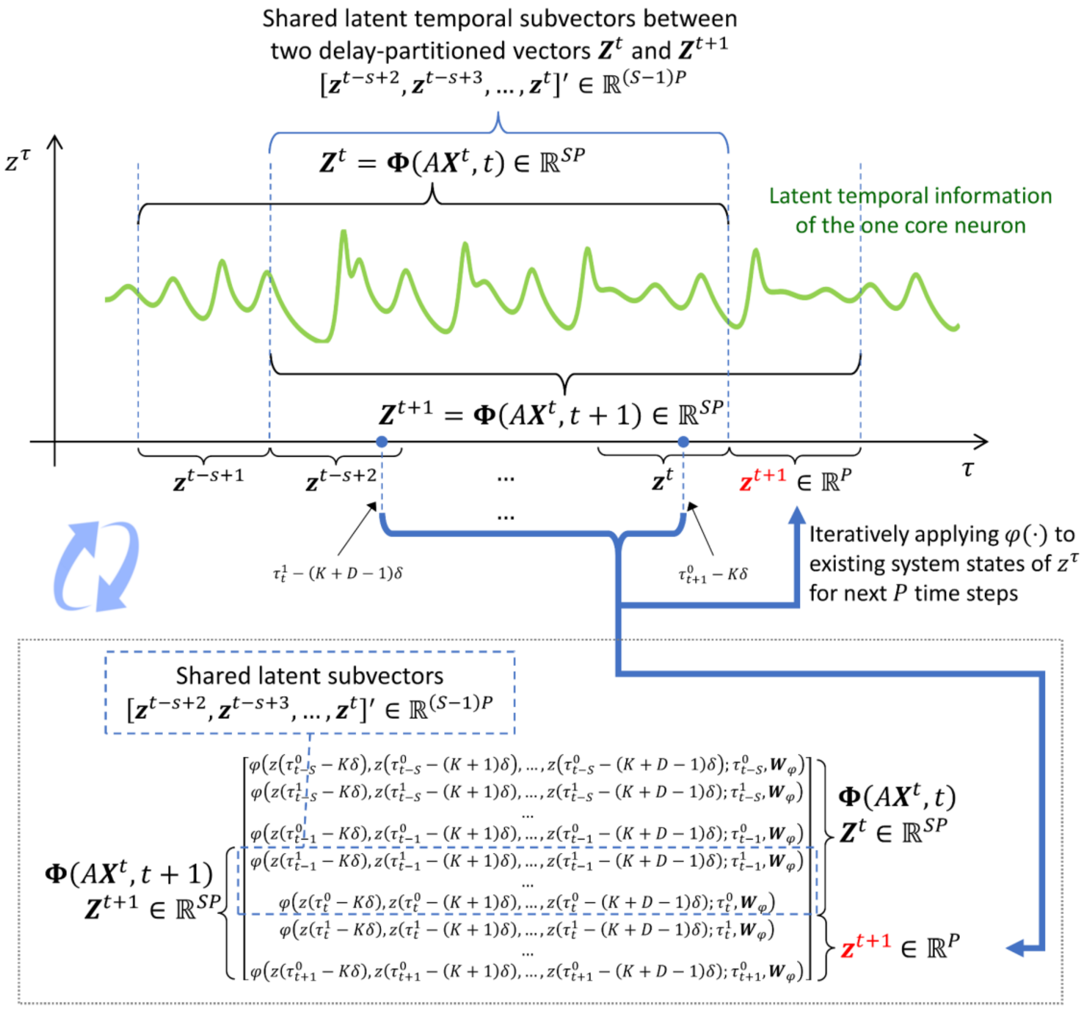

Figure S18. Schematic illustration of one step of the forward process in the OCN $\boldsymbol{\Phi}$. As the delay-partitioned vectors $\boldsymbol{Z}^{t+i}, i = 1,2, \dots$ are constructed through a delay-embedding scheme, it is noteworthy that two temporally adjacent delayed vectors share $S-1$ subvectors. For instance, $[\boldsymbol{z}^{t-s+2}, \boldsymbol{z}^{t-s+3}, \dots, \boldsymbol{z}^t]' \in \mathbb{R}^{(S-1)P}$ serves as the shared vector between $\boldsymbol{Z}^t$ and $\boldsymbol{Z}^{t+1}$, implying that advancing merely one step from $\boldsymbol{Z}^t$ to obtain $\boldsymbol{z}^{t+1} \in \mathbb{R}^P$ is sufficient for deriving $\boldsymbol{Z}^{t+1}$. Therefore, obtaining the P states of the system z^τ contained in temporal subvector $\boldsymbol{z}^{t+1} \in \mathbb{R}^P$ enables the construction of $\boldsymbol{Z}^{t+1}$. Considering that the state of the latent delay dynamical system z^τ is determined according to the delay difference equation $\varphi(\cdot)$ (Eq. (S12)), one step of the forward process of the OCN $\boldsymbol{\Phi}$ from time t to $t+1$ (i.e., from $\boldsymbol{Z}^t = \boldsymbol{\Phi}(A\boldsymbol{X}^t, t)$ to $\boldsymbol{Z}^{t+1} = \boldsymbol{\Phi}(A\boldsymbol{X}^t, t+1)$) essentially involves iteratively applying $\varphi(\cdot)$ to the existing states of the delay system z^τ for the next P latent time points, where

P is the partitioning factor between time t and the latent time τ. Extending this logic, the OCN $\mathbf{\Phi}$ has the ability to acquire more delayed vectors for future time points.

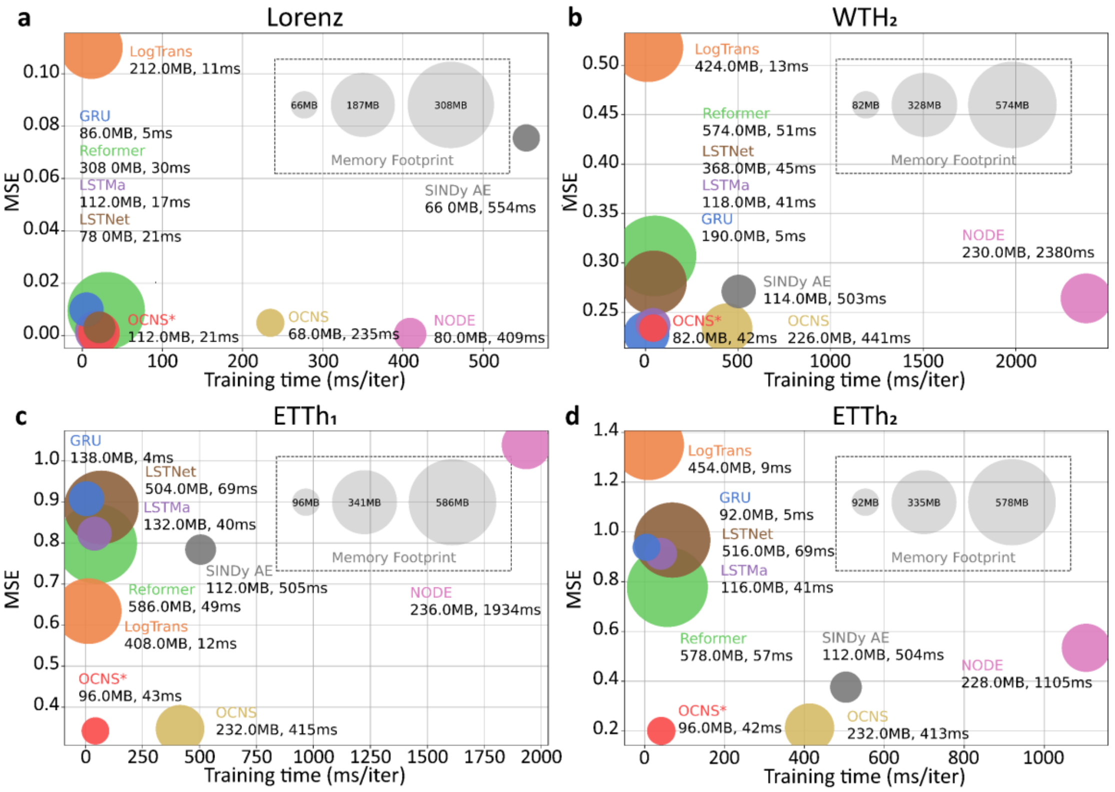

Figure S19. Model efficiency comparison under (a) Lorenz, (b) WTH_2, (c) $ETTh_1$, and (d) $ETTh_2$ datasets.

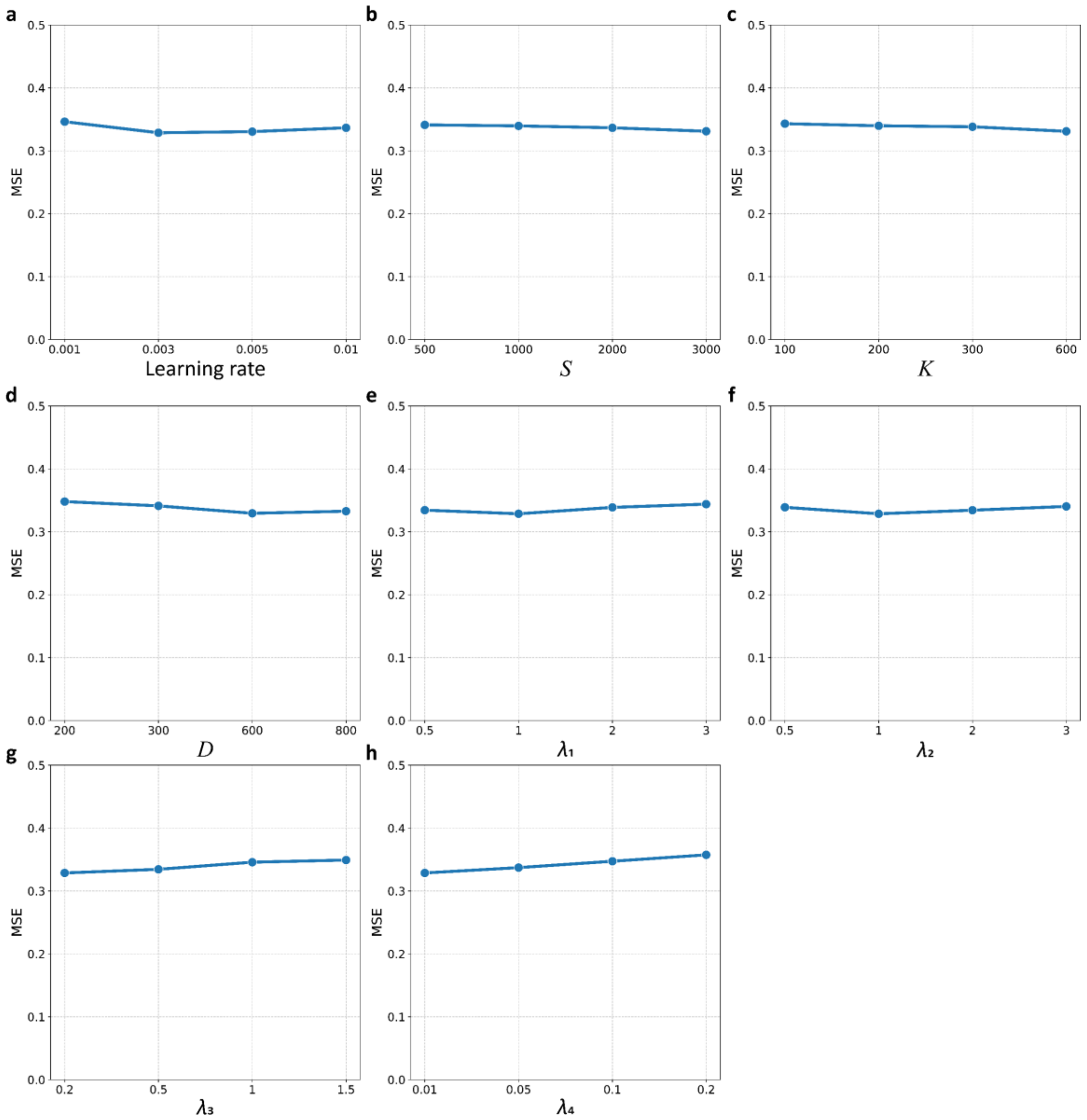

Figure S20. Hyperparameter sensitivity of OCNS on $ETTh_1$ dataset with respect to the learning rate (a), the delay-embedding dimension S (b), the step size of the first delay K (c), the number of temporal delays D (d) in the OCN module, and four loss component weights $\lambda_1 - \lambda_4$ (e-h).

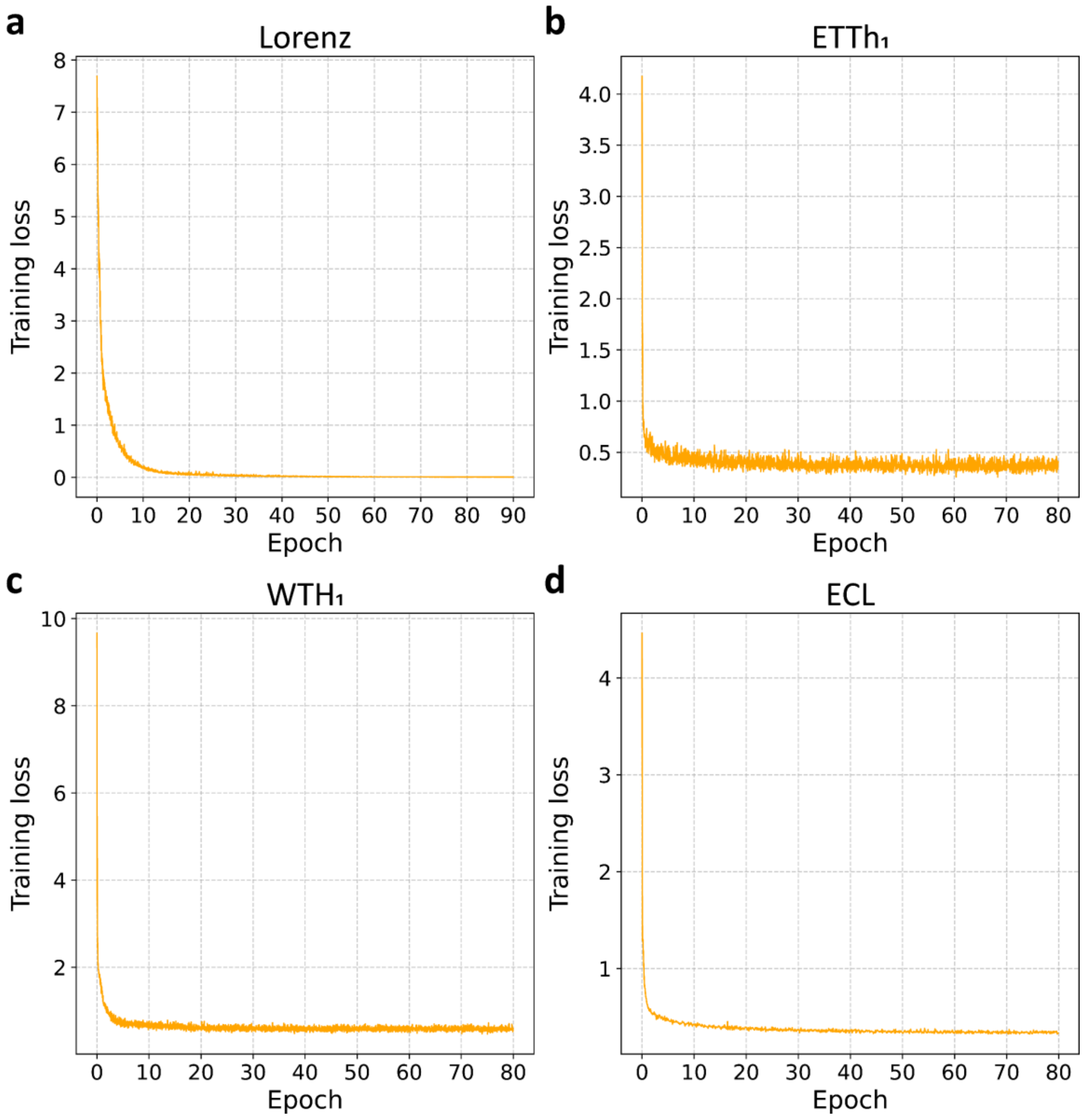

Figure S21. Convergence curves of the training loss for OCNS on the Lorenz (a), $ETTh_1$ (b), WTH_1 (c) and ECL (d) datasets.

Table S1. Results of an ablation study involving the exclusion of different loss terms from the fully supervised training loss, as defined in Eq. (S10). We report the MSE and MAE metrics, which are evaluated on the test set of the noise-free 9D coupled Lorenz dataset.

Metric	Without $\mathcal{L}_{\mathcal{EC}}$	Without $\mathcal{L}_{\mathcal{ED}}$	Without $\mathcal{L}_{\mathcal{R}}$	Full loss
MSE	2.7×10^{-3}	3.6×10^{-3}	3.2×10^{-3}	$\mathbf{1.4 \times 10^{-3}}$
MAE	4.0×10^{-2}	4.6×10^{-2}	4.6×10^{-2}	$\mathbf{2.7 \times 10^{-2}}$

Table S2. Prediction performance of OCNS across different data dimensions (n) for short-term high-dimensional systems. We report the MSE and MAE metrics on the Lorenz dataset ($\sigma = 0.5$).

System dimension n	Metric	OCNS	Informer	LogTrans	Reformer	GRU	LSTMa
10	MSE	1.304	2.795	2.887	2.941	1.989	2.045
	MAE	1.009	1.533	1.6	1.656	1.336	1.358
20	MSE	1.247	2.702	1.334	1.305	1.387	1.059
	MAE	1.05	1.535	1.094	1.08	1.102	0.955
30	MSE	0.997	2.036	1.015	1.445	1.492	1.448
	MAE	0.95	1.293	0.945	1.125	1.146	1.127
60	MSE	0.375	1.339	0.281	2.257	1.677	1.464
	MAE	0.539	1.031	0.508	1.469	1.244	1.178
90	MSE	0.156	1.217	0.446	1.952	1.359	1.665
	MAE	0.337	0.95	0.649	1.374	1.089	1.259

Table S3. Classification performance comparison between generalized OCNS versions with and without the reconstruction module on five benchmark classification datasets. The text in bold denotes the best result obtained in each row. The classification accuracy of the generalized OCNS surpasses that of its counterparts in most cases, especially when the length of the latent time series N is large. These results demonstrate the effectiveness of the multi-task learning approach within the generalized OCNS for the classification task.

**Dataset**	***N***	**Generalized OCNS**	**Generalized OCNS without reconstruction**
MNIST	100	**99.03%**	98.99%
	200	**99.12%**	98.95%
	400	**99.15%**	98.91%
	800	**99.29%**	98.86%
Fashion-MNIST	100	89.06%	**89.20%**
	200	**89.28%**	88.89%
	400	**89.35%**	89.34%
	800	**89.50%**	88.56%
CIFAR-10	150	60.39%	**60.92%**
	300	**60.92%**	60.18%
	600	**61.83%**	60.70%
	1200	**62.34%**	56.60%
CIFAR-100 (coarse labels)	150	**42.31%**	42.03%
	300	**42.73%**	42.71%
	600	**43.42%**	42.76%
	1200	**43.74%**	42.72%
Cropped version of SVHN	150	86.53%	**86.64%**
	300	**86.98%**	86.42%
	600	**87.06%**	87.01%
	1200	**87.13%**	87.07%

Table S4. Summary of the default parameters used by the OCNS for each forecasting dataset.

**Dataset**	Lorenz	$ETTh_1$	$ETTh_2$	$ETTm_1$	$ETTm_2$	ECL	WTH_1	WTH_2
λ_1	2.0	1.0	1.0	1.0	1.0	1.0	1.0	1.0
λ_2	2.0	1.0	1.0	3.0	3.0	1.0	1.0	1.0
λ_3	1.0	1.0	1.0	1.0	0.2	1.0	1.0	1.0
λ_4	0.05	0.1	0.1	0.1	0.1	0.1	0.1	0.1
S	480	1000	1000	800	1000	1200	1600	1000
K	60	200	200	400	500	300	800	200
D	400	300	300	800	800	700	400	300
m	20	48	48	48	48	96	48	48
l	8	24	24	24	24	48	24	24
**Learning rate**	5e-3	5e-3	5e-3	1e-3	1e-3	3e-3	3e-3	3e-3
**# of epochs**	90	80	80	80	80	80	80	80

Table S5. Summary of the dataset statistics for the benchmark. The *Dimension* indicates the number of variates/time series. The *Number of samples* denotes the total number of samples in the dataset, organized in (training, validation, testing).

Dataset	Dimension	Number of samples
Lorenz	9	24,000 / 3,000 / 3,000
$ETTh_1$, $ETTh_2$	7	8,569 / 2,857 / 2,857
$ETTm_1$, $ETTm_2$	7	34,489 / 11,497 / 11,497
WTH_1	12	20,089 / 7,177 / 7,177
Electricity	321	10,657 / 2,113 / 2,833
SMD	38	**566,653 / 141,658 / 655,846**
WTH_2	21	**315,289 / 525,37 / 523,94**

Table S6. Comparative overview of OCNS and time-delayed RC.

		OCNS	Time-delayed RC
Input		AX^t as the initial temporal states of OCNS	$\boldsymbol{X}^t$
Dynamic Evolution Process	System Type	**Autonomous system**	**Non-autonomous system**
	Driven Function	$z^t = \sigma(w_0 + \sum_{j=1}^{D} w_j z^{t-(K-1+j)})$	$\boldsymbol{r}^t = \alpha \boldsymbol{r}^{t-1} + (1-\alpha)\sigma(W_{in}\boldsymbol{X}^t + W_{res}\boldsymbol{r}^{t-1} + \boldsymbol{b})$
	State Evolution	Based on historical states, with no dependence on external inputs.	Influenced by both previous states and an external input vector $\boldsymbol{X}^t$.
Output		$\boldsymbol{Y}^t = B\boldsymbol{Z}^t$, where $\boldsymbol{Z}^t = (z^{t-S+1}, z^{t-S+2}, \ldots, z^t)'$, S denotes the delay-embedding dimension.	$\boldsymbol{Y}^t = W_{out}\widetilde{\boldsymbol{R}}^t$, where $\widetilde{\boldsymbol{R}}^t = (r_1^t, r_1^{t-1}, \ldots, r_1^{t-(d_1-1)}, r_2^t, r_2^{t-1}, \ldots, r_2^{t-(d_2-1)}, \ldots, r_q^t, r_q^{t-1}, \ldots, r_q^{t-(d_q-1)})'$, i.e., each neuron r_i contributes d_i lagged dynamics to $\widetilde{\boldsymbol{R}}^t$.
Parameters	Input Matrix	A (Requires training)	W_{in} (No training required)
	Weight Matrix	$W = \{w_j \mid j = 0, 1, \ldots, D\}$ (Requires training)	W_{res} (No training required)
	Output Matrix	B (Requires training)	W_{out} (Requires training)

Table S7. Multivariate forecasting results obtained on the numerical Lorenz datasets and real-world datasets by the OCNS and other methods.

Dataset	Metric	OCNS	OCNS+	Time-LLMa	Time-LLMb	Informer	LogTrans	Reformer	GRU	LSTMa	LSTNet	NODE	SINDy AE
Lorenz ($\sigma = 0$)	MSE	0.00489	0.000781	0.000878	0.00165	0.00471	0.110	0.00950	0.00997	0.00159	0.00318	**0.000630**	0.0755
	MAE	0.0497	0.0209	**0.0161**	0.0308	0.0508	0.226	0.0698	0.0724	0.0282	0.0433	0.0184	0.193
Lorenz ($\sigma = 0.5$)	MSE	0.00828	**0.00460**	0.0103	0.0102	0.00849	0.0740	0.0138	0.0191	0.00945	0.00693	0.00709	0.0528
	MAE	0.0704	**0.0538**	0.0780	0.0780	0.0713	0.201	0.0910	0.106	0.0760	0.0657	0.0670	0.165
Lorenz ($\sigma = 1.0$)	MSE	0.0192	**0.0158**	0.0303	0.0277	0.0192	0.0870	0.0380	0.0330	0.0223	0.0190	0.0259	0.106
	MAE	0.110	**0.100**	0.135	0.130	0.110	0.218	0.152	0.142	0.118	0.109	0.128	0.238
$ETTh_1$	MSE	0.347	0.319	0.334	**0.307**	0.577	0.634	0.798	0.908	0.823	0.887	1.039	0.784
	MAE	0.385	0.370	0.372	**0.356**	0.549	0.580	0.664	0.693	0.678	0.727	0.757	0.517
$ETTh_2$	MSE	0.213	0.198	0.185	**0.182**	0.535	1.349	0.778	0.938	0.912	0.967	0.534	0.376
	MAE	0.290	0.283	0.271	**0.267**	0.562	0.951	0.625	0.755	0.729	0.726	0.530	0.452
WTH_1	MSE	0.368	0.344	0.387	0.370	**0.335**	0.534	0.513	0.388	0.416	0.483	0.389	0.412
	MAE	0.401	**0.372**	0.405	0.391	0.381	0.537	0.498	0.407	0.410	0.451	0.426	0.344
WTH_2	MSE	0.234	**0.219**	0.279	-	0.295	0.518	0.307	0.228	0.237	0.280	0.264	0.271
	MAE	0.226	**0.209**	0.236	-	0.300	0.455	0.349	0.249	0.265	0.223	0.271	0.289
ECL	MSE	0.230	0.215	**0.185**	-	0.538	0.602	0.565	0.493	0.503	0.492	1.163	0.722
	MAE	0.328	0.316	**0.281**	-	0.506	0.516	0.520	0.449	0.463	0.455	0.870	0.617
$ETTm_1$	MSE	0.567	**0.400**	0.521	0.493	0.463	0.764	0.772	0.671	0.716	0.663	0.706	0.802
	MAE	0.467	**0.392**	0.430	0.418	0.438	0.606	0.602	0.565	0.572	0.553	0.549	0.560
$ETTm_2$	MSE	0.129	**0.120**	0.128	0.128	0.177	0.674	0.439	0.586	0.463	0.574	0.415	0.311
	MAE	0.230	**0.218**	0.227	0.226	0.305	0.641	0.490	0.583	0.499	0.557	0.440	0.361
SMD	MSE	0.226	0.218	**0.205**	-	0.313	0.382	0.278	0.522	0.501	0.458	0.400	0.465
	MAE	0.123	0.116	**0.114**	-	0.170	0.194	0.156	0.257	0.239	0.226	0.190	0.202
**1st Count**		0	11	5	4	1	0	0	0	0	0	1	0

[1] We highlight the best performances obtained in each row in bold.

[2] Time-LLMa and Time-LLMb denote the Time-LLM method with GPT-2 [9] and LlaMA-7B [10] backbones, respectively.

[3] "-" indicates that the result could not be obtained because the training time exceeded the 24-hour time limit.

Table S8. Comparison of parameter requirements between the OCNS and ten other methods on real-world datasets, with comparable or superior OCNS performances as shown in Table S7.

Dataset	Number of parameters ($\times 10^6$)										
	OCNS	OCNS+	Time-LLM[a]	Time-LLM[b]	Informer	LogTrans	Reformer	GRU	LSTMa	LSTNet	NODE	SINDy AE
$ETTh_1$	**0.025**	0.036	177.114	6651.987	11.330	5.567	5.786	1.933	1.303	0.682	1.016	0.764
$ETTh_2$	**0.012**	0.082	177.114	6651.987	11.330	5.567	5.786	1.090	1.303	0.682	1.016	0.764
WTH_1	**0.037**	0.088	175.304	6642.510	19.492	5.781	5.791	1.093	1.307	0.695	1.026	0.770
WTH_2	**0.033**	0.063	175.304	6642.510	11.375	6.177	5.801	4.357	0.965	0.587	1.044	0.842
ECL	**0.695**	0.731	175.313	6642.519	12.456	26.889	6.112	4.717	1.614	1.366	1.156	1.141
$ETTm_1$	**0.019**	0.065	177.114	6651.987	11.330	5.567	5.786	1.933	1.303	0.682	1.016	0.826
$ETTm_2$	**0.021**	0.060	177.114	6651.987	11.330	5.567	5.786	1.933	1.303	0.682	1.016	0.764
SMD	**0.058**	0.095	175.304	6642.510	19.587	6.960	5.818	4.377	0.978	0.622	1.078	0.801
Average percentage	/	49.86%	0.064%	0.0017%	0.88%	0.75%	1.86%	3.08%	7.64%	10.30%	9.98%	10.85%

[1] *Average percentage* indicates the proportion of the parameters used by the OCNS relative to the other methods. The text in bold denotes the minimum number of parameters obtained in each row.

[2] Time-LLM[a] and Time-LLM[b] denote the Time-LLM method with GPT-2 [9] and LlaMA-7B [10] backbones, respectively.

Table S9. Multivariate forecasting results obtained on real-world datasets by the OCNS and other comparative methods with the same parameter scale.

Dataset	Metric	OCNS	Informer*	LogTrans*	Reformer*	GRU*	LSTMa*	LSTNet*	NODE*	SINDy AE*
ETTh$_1$	MSE	**0.347**	0.817 (+42%)	1.147 (+81%)	1.143 (+43%)	1.125 (+24%)	0.902 (+10%)	1.251 (+41%)	1.985 (+91%)	1.237 (+58%)
	MAE	**0.385**	0.650 (+18%)	0.806 (+39%)	0.810 (+22%)	0.797 (+15%)	0.687 (+1%)	0.869 (+20%)	1.019 (+35%)	0.609 (+18%)
ETTh$_2$	MSE	**0.213**	1.068 (+100%)	3.005 (+123%)	1.13 (+45%)	1.302 (+39%)	1.249 (+37%)	1.127 (+17%)	0.857 (+60%)	0.391 (+4%)
	MAE	**0.290**	0.852 (+52%)	1.330 (+40%)	0.755 (+21%)	0.837 (+11%)	0.856 (+17%)	0.823 (+13%)	0.71 (+34%)	0.463 (+2%)
WTH$_1$	MSE	**0.368**	0.517 (+54%)	0.723 (+35%)	0.598 (+17%)	0.463 (+19%)	0.465 (+12%)	0.615 (+27%)	0.401 (+3%)	0.589 (+43%)
	MAE	**0.401**	0.518 (+36%)	0.666 (+24%)	0.589 (+18%)	0.492 (+21%)	0.503 (+23%)	0.533 (+18%)	0.438 (+3%)	0.473 (+38%)
WTH$_2$	MSE	**0.234**	0.341 (+16%)	0.663 (+28%)	0.456 (+49%)	0.342 (+50%)	0.406 (+71%)	0.636 (+127%)	0.366 (+39%)	0.338 (+25%)
	MAE	**0.226**	0.344 (+15%)	0.556 (+22%)	0.437 (+25%)	0.361 (+45%)	0.385 (+45%)	0.456 (+104%)	0.312 (+15%)	0.298 (+3%)
ECL	MSE	**0.230**	0.941 (+75%)	0.999 (+66%)	1.471 (+160%)	1.415 (+187%)	1.497 (+198%)	0.624 (+27%)	1.315 (+13%)	0.946 (+31%)
	MAE	**0.328**	0.798 (+58%)	0.731 (+42%)	0.897 (+72%)	0.969 (+116%)	0.982 (+112%)	0.560 (+23%)	0.917 (+5%)	0.702 (+14%)
ETTm$_1$	MSE	**0.567**	1.424 (+208%)	1.298 (+70%)	0.986 (+28%)	1.149 (+71%)	1.064 (+49%)	1.609 (+143%)	1.69 (+139%)	1.868 (+133%)
	MAE	**0.467**	0.958 (+119%)	0.86 (+42%)	0.738 (+23%)	0.812 (+44%)	0.799 (+40%)	1.020 (+84%)	0.983 (+79%)	0.585 (+4%)
ETTm$_2$	MSE	**0.129**	0.362 (+105%)	0.803 (+19%)	0.671 (+53%)	0.758 (+29%)	0.707 (+53%)	0.765 (+33%)	0.629 (+52%)	0.679 (+118%)
	MAE	**0.230**	0.436 (+43%)	0.707 (+10%)	0.661 (+35%)	0.619 (+6%)	0.655 (+31%)	0.653 (+17%)	0.616 (+40%)	0.603 (+67%)
SMD	MSE	**0.226**	0.468 (+50%)	0.515 (+35%)	0.455 (+64%)	0.915 (+75%)	0.615 (+23%)	0.748 (+63%)	1.133 (+183%)	0.499 (+7%)
	MAE	**0.123**	0.244 (+44%)	0.335 (+73%)	0.182 (+17%)	0.522 (+103%)	0.364 (+52%)	0.448 (+98%)	0.654 (+244%)	0.259 (+28%)
**1st Count**		**16**	0	0	0	0	0	0	0	0

* A method name denoted by an asterisk represents a reduced-parameter counterpart with respect to the original method. The percentages in parentheses indicate the forecasting performance degradations that occur when using fewer parameters than that employed by the corresponding original method. The text in bold denotes the best performances obtained in each row.

Table S10. Performance comparisons conducted on classification tasks with respect to different time points N in the latent one-dimensional system. The text in bold denotes the best results obtained in each row.

Dataset	*N*	OCNS	Fit-DNN	MLP
MNIST	100	**99.03%**	97.31%	98.33%
	200	**99.12%**	98.49%	98.32%
	400	**99.15%**	98.91%	98.11%
	800	**99.29%**	98.97%	98.11%
Fashion-MNIST	100	**89.06%**	86.61%	88.69%
	200	**89.28%**	87.82%	88.83%
	400	**89.35%**	88.59%	88.14%
	800	**89.50%**	89.18%	88.39%
CIFAR-10	150	**60.39%**	48.29%	57.51%
	300	**60.92%**	51.42%	57.73%
	600	**61.83%**	53.94%	58.33%
	1200	**62.34%**	54.99%	58.14%
CIFAR-100 (coarse label)	150	**42.31%**	29.39%	39.39%
	300	**42.73%**	32.73%	40.41%
	600	**43.42%**	34.51%	40.77%
	1200	**43.74%**	35.41%	41.51%
Cropped version of SVHN	150	**86.53%**	73.45%	85.34%
	300	**86.98%**	78.93%	85.59%
	600	**87.06%**	80.85%	85.54%
	1200	**87.13%**	81.38%	85.41%

3. Supplementary Sections

Section S1. Enhancements to the modules in the OCNS

In the main text, the OCNS predominantly employs linear transformations, with most of the nonlinear representation capabilities provided by a very simple structured nonlinear OCN $\mathbf{\Phi}$. This design choice was made to keep the model as compact and straightforward as possible. However, such simplicity might limit the performance of model to represent complex nonlinear systems effectively. Therefore, we propose several enhancements to improve the OCNS's representation capabilities:

Incorporating 'sub-time' to the one-dimensional system. Inspired by the generalized embedding theory [1,11] and time multiplexing technique [12], we present such a scheme of OCNS by adopting latent time τ as the 'sub-time' of real time t, as shown in Figure S2. For time t, within one incremental unit (time interval) of length 1, we define P equidistant points separated in time with sub-interval $\delta = 1/P$ and $P > 2$. All these points extended from the real time points of t are defined as 'sub-time' τ with a form anchored to the real time t:

$$\tau_t^j = t + j\delta, \tag{S11}$$

where $j = 0,1,\ldots,P-1$ is the position of 'sub-time' within two adjacent real time points t and $t+1$. With this setting, we consider a one-dimensional system $z(\tau)$ (rather than $z(t)$ in the main manuscript) that evolves in the latent discrete-time τ with a small temporal increment δ and is governed by a delay difference equation (DDE) [12–15] incorporating multiple temporal delays:

$$z(\tau_t^j) = \varphi\big(z(\tau_t^j - K\delta), z(\tau_t^j - (K+1)\delta), \ldots, z(\tau_t^j - (K+D-1)\delta); \tau_t^j, W_\varphi\big) \tag{S12}$$

where $K\delta, (K+1)\delta, \ldots, (K+D-1)\delta$ denote the time delays, K is a constant representing the step size of the first delay, D is the total number of delays, W_φ indicates the parameters in the difference function $\varphi(\cdot)$. Similarly, for the sub-time τ, we denote $z(\tau)$ as z^τ for convenience. Figure S2b shows how the OCN architecture $\mathbf{\Phi}$ processes the iterative acquisition of the inner delayed dynamics for z^τ within the

'sub-time' τ. For this, the input state $\boldsymbol{X}^t$ of any dimension n is multiplied by a mask (the input weight $A \in \mathbb{R}^{L \times n}$), resulting in a temporal input stream $\boldsymbol{J}^t = A\boldsymbol{X}^t \in \mathbb{R}^L$ (Figure S2a) that is fed into the OCN $\boldsymbol{\Phi}$ as the initial states. To represent the whole states of a high-dimensional dynamical system $\boldsymbol{X}^t$, we define a delay-partitioned vector $\boldsymbol{Z}^t = \boldsymbol{\Phi}(\boldsymbol{J}^t, t) = \boldsymbol{\Phi}(A\boldsymbol{X}^t, t) = [\boldsymbol{z}^{t-S+1}, \boldsymbol{z}^{t-S+2}, \dots, \boldsymbol{z}^t]' \in \mathbb{R}^{SP}$ which is presented in Figure S2a, where row vector $\boldsymbol{z}^{t-S+k} = \left[z^{\tau^1_{t-S+k-1}}, z^{\tau^2_{t-S+k-1}}, \dots, z^{\tau^{P-1}_{t-S+k-1}}, z^{\tau^0_{t-S+k}}\right] \in \mathbb{R}^P$ $(k = 1,2, \dots, S)$ represents a latent temporal subvector, and S denotes the delay-embedding dimension on time t. Therefore, the delay-partitioned vector is constructed in the form of $\boldsymbol{Z}^t = \left[z^{\tau^1_{t-S}}, z^{\tau^2_{t-S}}, \dots, z^{\tau^0_t}\right]' \in \mathbb{R}^{SP}$, being a long-delayed temporal sequence that contains the dynamics of z^τ corresponding to the real time t. We have also demonstrated that, with the incorporation of 'sub-time' τ, the OCN $\boldsymbol{\Phi}$ remains a generic embedding of the original nonlinear dynamical system (Section S2). To more concretely illustrate the delay dynamical system in Eq. (S12), the dynamical evolution process of z^τ governed by the difference function $\varphi(\cdot)$ is presented in Figure S3. As an example of one-step forward forecasting, we provide a comprehensive workflow of the OCN $\boldsymbol{\Phi}$ with 'sub-time' τ in Figure S18. In fact, the OCNS framework with the introduction of 'sub-time' seamlessly aligns with the original objectives and requires only minimal modifications, such as replacing all latent states in the vanilla OCNS (e.g., z^t) with latent temporal vectors (e.g., $\boldsymbol{z}^t$).

Neural network module for enhanced approximation of nonlinear difference function $\varphi(\cdot)$. Despite the dynamics of the delay dynamical system z^t derived from the OCN $\boldsymbol{\Phi}$ being confined within a single latent variable, the delayed feedback in this system introduces significant complexity and potent representational capabilities. Nevertheless, the approximation of the difference function $\varphi(\cdot)$ (Eq. (1) in the main manuscript) in the OCN $\boldsymbol{\Phi}$ plays a crucial role for representing/reconstructing the original complex dynamic system. Several feasible approaches are available for the implementation of $\varphi(\cdot)$, except for only using the only one-neuron model ((Eq. (2) in the main text). For example, the practical implementation of function $\varphi(\cdot)$ can take the form of any RNN that processes delayed feedback signals as input and outputs the

subsequent state of the one-dimensional delay system. In order to improve the nonlinear representation ability of the OCN $\mathbf{\Phi}$, we can adopt a small temporal convolutional network (TCN) [16] for emulating $\varphi(\cdot)$ to capture the dynamics in the delay system. The decision to use a TCN rather than canonical RNNs is grounded in several advantages offered by the TCN, such as its longer effective memory length, flexible receptive field size, stable gradients, lower memory requirement for training, adaptability to variable-length inputs, and enhanced parallelism [16]. Most importantly, the causal convolution mechanism in the TCN enables it to generate a sequence of multiple future states in one forward operation rather than in a step-by-step manner, which drastically improves the speed of forecasting.

Replacing linear mapping B with more powerful decoding methods. A powerful decoder can significantly enhance the model's performance. Therefore, we can replace the linear mapping B with nonlinear decoders, such as a compact transformer [17] encoder.

Section S2. Dynamical systems and delay embedding theory

Generally, the dynamics of a discrete-time dissipative system can be formed as,

$$\boldsymbol{X}^{t+1} = \phi(\boldsymbol{X}^{t}), \tag{S13}$$

Where $\phi: \mathbb{R}^n \to \mathbb{R}^n$ denotes a nonlinear map, whose n-dimensional variables are denoted as vector $\boldsymbol{X}^t = [x_1^t, x_2^t, \dots, x_n^t]'$ with the time superscript t and the vector transpose symbol " $'$ ". After a sufficient time, all of the states converge into a compact manifold $\mathcal{M}$ with the box-counting dimension $boxdim(\mathcal{M}) = d_{\mathcal{M}}$. Denoting the attractor contained in manifold $\mathcal{M}$ as $\mathcal{A}$ with the box-counting dimension d, the delay embedding theorem indicates that only using observed long-term data of a single variable can topologically reconstruct the attractor $\mathcal{A}$ of the original high-dimensional system when certain conditions are satisfied. We introduce the Takens' embedding theorem [2] first.

Theorem 1. Let $\mathcal{M}$ be a compact manifold of box-counting dimension $d_{\mathcal{M}}$. For pairs

$(\Psi, h), \Psi: \mathcal{M} \rightarrow \mathcal{M}$ a smooth (at least $\mathbb{C}^2$) diffeomorphism and $h: \mathcal{M} \rightarrow \mathbb{R}$ a smooth (at least $\mathbb{C}^2$) function, it is a generic property that the map $\Upsilon_{\Psi,h}: \mathcal{M} \rightarrow \mathbb{R}^K$, defined by

$$\Upsilon_{\Psi,h}(X) = \left[h(X), h\big(\Psi(X)\big), \dots, h\big(\Psi^{K-1}(X)\big)\right]' \tag{S14}$$

is an embedding when the integer $K > 2d_{\mathcal{M}}$.

In fact, the dimension of the original system or the manifold $\mathcal{M}$ is usually much larger than that of attractor $\mathcal{A}$, i.e., $d_{\mathcal{M}} \gg d$, which limits the application scope of the embedding theorem. Thus, Sauer *et al.* [1] generalized the embedding theorem for fractal dimension, i.e., the map defined in Theorem 1 is still one-to-one on $\mathcal{A}$ and immersions on each compact subset of a smooth manifold contained in $\mathcal{A}$ as long as $K > 2d$.

Theorem 2. Let h be a diffeomorphism on a compact attractor $\mathcal{A}$ of $\mathbb{R}^n$ with $\text{boxdim}(\mathcal{A}) = d$, and let $K > 2d$ be an integer. Assume that for every positive integer $u \leq K$, the set $\mathcal{A}_u$ of periodic points of period u satisfies $\text{boxdim}(\mathcal{A}_u) < u/2$, and that the linearization $D\Psi^u$ for each of these orbits has distinct eigenvalues. Then for almost every smooth function h on $\mathcal{A}$, the delay coordinate map $\Upsilon_{\Psi,h}: \mathcal{A} \rightarrow \mathbb{R}^K$ is:

1. One-to-one on $\mathcal{A}$.
2. An immersion on each compact subset C of a smooth manifold contained in $\mathcal{A}$.

Remark 1. The proof of Theorem 2 is easily extended to the more general case where the reconstruction map Υ consists of a mixture of lagged observations. The more general result says that

$$\Upsilon(X) = \left[h_1(X), \dots, h_1\big(\Psi^{K_1-1}(X)\big), \dots, h_q(X), \dots, h_q\big(\Psi^{K_q-1}(X)\big)\right]' \tag{S15}$$

satisfies the conclusions of Theorem 2 as long as $\sum_{i=1}^{q} K_i > 2d$ and the corresponding conditions on the periodic points are satisfied. Those conditions are that $\text{boxdim}(\mathcal{A}_u) < u/2$ for $u \leq \max\{K_1, \dots, K_q\}$.

In particular, for the situation where 'sub-time' τ is introduced, letting $X = \boldsymbol{X}^t$, $\Psi = \phi$, $q = P$, $K_i = S\ (i = 1,2,\dots,P)$, and $h_i(\phi^j(\boldsymbol{X}^t)) = z^{\tau_{t-S+j}^{i-1}} \in \mathbb{R}\ (i = 1,2,\dots,P; j = 1,2,\dots,S)$, where $z^{\tau_{t-S+j}^{i-1}}$ denotes the states derived from a latent one-dimensional delay dynamical system z^τ, the map above (Eq. (S15) has the following form with $\Upsilon: \mathcal{A} \to \mathbb{R}^{SP}$ and

$$\Upsilon(\boldsymbol{X}^t) = \left[z^{\tau_{t-S}^1}, \dots, z^{\tau_{t-S+1}^0}, \dots, z^{\tau_{t-1}^1}, \dots, z^{\tau_t^0}\right]' = [\boldsymbol{z}^{t-S+1}, \dots, \boldsymbol{z}^t]' = \boldsymbol{Z}^t, \qquad \text{(S16)}$$

with row vector $\boldsymbol{z}^{t-S+k} = \left[z^{\tau_{t-S+k-1}^1}, z^{\tau_{t-S+k-1}^2}, \dots, z^{\tau_{t-S+k-1}^{P-1}}, z^{\tau_{t-S+k}^0}\right] \in \mathbb{R}^P, k = 1,2,\dots,S$. In this work, Eq. (S16) is used in the OCNS-based STI equations as the primary equation (main text Eq. (3)).

Section S3. Coupled Lorenz system

The numerical dataset used to validate the forecasting performance of the OCNS consists of a synthetic 9D coupled Lorenz system [18]. The system is composed of 3 subsystems, with each subsystem formed as follows:

$$\begin{cases} \dot{x}_i = \sigma(y_i - x_i) + cx_{i-1} \\ \dot{y}_i = \rho x_i - y_i - x_i z_i \\ \dot{z}_i = x_i y_i - \beta z_i \end{cases}, \qquad \text{(S17)}$$

where $i = 1,2,3$ denotes the i-th subsystem, cx_{i-1} is the coupled factor for coupling the i-th subsystem with the previous subsystem, and we set $i - 1 = 3$ for the situation in which $i = 1$ to keep the system closed; $c = 0.1, \rho = 28, \beta = \frac{8}{3}, \sigma = 10$ are constants for each subsystem. In the implementation of the coupled Lorenz system, the initial values of all variables are assigned based on a normal distribution. The time interval is set as $\Delta t = 0.02$, and data collection occurs after the transient dynamics stabilize. In this study, the OCNS exclusively utilizes the generated time-course datasets derived from Eq. (S17) to illustrate the forecasting process of the temporal evolution procedure. Notably, Eq. (S17) does not contribute any prior knowledge in this forecasting process. For this dataset, $m = 20$ time points are used as the known series, and the subsequent $l = 8$ steps of multivariate dynamic data are forecasted.

Section S4. Brief introduction to the real-world forecasting benchmark datasets

In this work, we preprocessed each dataset using a sliding window approach with an input length of m and a prediction length of l, where the window moves with a step size of 1 to generate time series samples. The statistics for all datasets are presented in Table S5.

Electricity transformer temperature (ETT) datasets. As a crucial indicator of long-term electric power deployment, it is important to understand and predict the dynamics of ETT and its associated variables. The ETT data utilized in this study were gathered from two distinct counties in China during the period from 2016.07 to 2018.07 [19]. Consistent with the data segmentation approach employed in Informer [19], the collected ETT data were divided into four datasets, namely, $ETTh_1$, $ETTh_2$, $ETTm_2$, and $ETTm_1$, based on different time intervals. Specifically, the $ETTh_1$ and $ETTh_2$ datasets operate at the 1-hour level, while $ETTm_1$ and $ETTm_2$ operate at the 15-minute level. The subscripts 1 and 2 correspond to different counties. Each dataset is composed of 7 variables ($n = 7$), encompassing high useful load (HUFL), high useless load (HULL), medium useful load (MUFL), medium useless load (MULL), low useful load (LUFL), low useless load (LULL), and oil temperature (OT). Each dataset is partitioned into training, validation, and test sets with a temporal distribution of 15/3/4 months. All the ETT datasets are available for download from https://github.com/zhouhaoyi/ETDataset.

Electricity consumption load (ECL) dataset. The original ECL dataset (https://archive.ics.uci.edu/ml/datasets/ElectricityLoadDiagrams20112014) comprises electricity consumption (Kwh) data recorded at 15-minute intervals for a total of 321 clients ($n = 321$) [20]. Adopting the preprocessing procedure outlined in Informer [19], we aggregate the original dataset based on every 4 points, resulting in an hourly ECL dataset from 2012.01 to 2013.12, to alleviate the impact of missing values. The dataset is partitioned into training, validation, and test sets with a temporal distribution of

15/3/4 months.

Weather datasets. The climate system is considered a complex dynamical system characterized by its intricate and chaotic nature, exhibiting recurrent large-scale configurations intertwined with essential physical properties. This complexity poses difficulties and challenges in terms of weather forecasting and climate modeling. In this work, we introduce two weather datasets, namely, WTH_1 and WTH_2, to validate the forecasting performance of the OCNS. The WTH_1 dataset encompasses a four-year period of data for nearly 1,600 locations in the United States (https://www.ncei.noaa.gov/data/local-climatological-data/), recorded hourly from the years 2010 to 2013. Each data point in the WTH_1 dataset consists of $n = 12$ climate features, including "wet bulb" and 11 other variables. For the WTH_1 dataset, we allocate the data into training, validation, and testing sets, with a split of 28/10/10 months, respectively. On the other hand, the WTH_2 dataset comprises $n = 21$ meteorological indicators recorded every 10 minutes at the Beutenberg meteorological station in Germany (https://www.bgc-jena.mpg.de/wetter/). For our experiments, we select the complete data for the years from 2016 to 2023. To establish the training, validation, and testing sets, we arrange the data in chronological order and apply a ratio of 7:1:2.

Server machine dataset (SMD). The SMD [21] is a 5-week-long dataset originally collected from a large Internet company and made publicly available on GitHub (https://github.com/NetManAIOps/OmniAnomaly/tree/master). It was initially designed for anomaly detection, with domain experts labeling anomalies and their corresponding dimensions in the testing set based on incident reports. The dataset was originally split into two equal subsets, with the first half used for training and the second half for testing. For our time series forecasting task, we preprocessed the dataset by filtering the testing set to exclude samples containing anomalous time points, retaining only normal data for analysis. Additionally, a small portion of the original training set was partitioned to serve as a validation set, as detailed in Table S5.

Section S5. Ablation study concerning the loss terms for forecasting

We evaluate the impacts of the different loss terms used in the fully supervised training process, as defined in Eq. (S10), on the performance achieved in multivariate time-series forecasting tasks. The ablation study is conducted using the noise-free 9D coupled Lorenz system. As illustrated in Table S1, the OCNS trained with the full loss achieves the best prediction performance, as evidenced by its lowest MSE and MAE values, which suggests that all the loss terms contribute significantly to reconstructing the original dynamical system within a latent one-dimensional delay dynamical system and facilitating accurate forecasting.

Section S6. Evaluating the OCNS on short-term time series across different data dimensions

Based on STI transformation, the OCNS is capable of fully exploiting spatial information from the input time series, especially in scenarios with short-term high-dimensional datasets. In this experiment, the OCNS is trained on time series obtained from the noisy ($\sigma = 0.5$) Lorenz system (Eq. (S17)) with different dimensions consisting of only 400 time points. To eliminate the influence of increased dimensional complexity on the evaluation of prediction performance, the OCNS is tasked with predicting one common target variable rather than all variables. As shown in Table S2, our proposed OCNS outperforms the majority of baseline models in most scenarios, and achieves better performance with higher-dimensional data, thereby validating the effectiveness of the STI transformation.

Section S7. Generalization of time-series learning framework for categorization

To delve deeper into the potential and capacity of time-series learning, we generalize the OCNS by incorporating a simple classifier for an image classification task. As the framework illustrated in Figure S4 shows, the OCNS retains its autoencoder structure,

with a k -classification classifier utilizing the latent time series as input for classification purposes. Therefore, the generalized framework embodies a multi-task learning approach and derives significant advantages from this strategy, including a robustness enhancement and better performance [22]. The effectiveness of multi-task learning for the time-series learning process in this study is further evidenced by the comprehensive analysis presented in Table S3.

For the classification task, the OCNS undergoes an end-to-end training procedure akin to that of the forecasting task. During this process, optimization not only extends the OCN $\mathbf{\Phi}$, the input weight matrix A, and the output weight matrix B but also involves determining the classification weight matrix W_c in the classifier. Notably, the delay-embedding scheme is not used in this context. Instead, the collaboration between the OCN $\mathbf{\Phi}$ and A facilitates the direct transformation of the spatial information $\boldsymbol{X}$ into a time series $\boldsymbol{Z}$ of a single latent variable, as depicted in Figure S4. Moreover, the output layer is configured with a size C equal to the number of classes to be categorized, i.e., the output vector $\boldsymbol{O} = [o_1, o_2, \dots, o_C] \in \mathbb{R}^C$. We choose the softmax function as the activation function, which assigns a probability distribution to each class. Consequently, the final output vector of the classifier is denoted as $\widehat{\boldsymbol{O}} = [\hat{o}_1, \hat{o}_2, \dots, \hat{o}_C] \in \mathbb{R}^C$, where each element

$$\hat{o}_i = \mathrm{softmax}(\boldsymbol{O}) = \frac{\exp(o_i)}{\sum_{j=1}^{C} \exp(o_j)}, \qquad i = 1,2, \dots C \tag{S18}$$

represents the probability that the input data point belongs to class i. Following this step, for N examples, the cross-entropy loss $\mathcal{L}_{CE}$ in the context of C-classification is computed as:

$$\mathcal{L}_{CE} = -\frac{1}{N} \sum_{i=1}^{N} \sum_{j=1}^{C} y(i,j) \log(\hat{o}_j). \tag{S19}$$

Here, $y(i,j)$ denotes the signal function and is defined as

$$y(i,j) = \begin{cases} 1, & \text{if the label of sample } i \text{ is class } j \\ 0, & \text{otherwise} \end{cases}. \tag{S20}$$

Since the generalized OCNS for classification is implemented in a multi-task learning manner, we train the model with the combined loss, which is formulated as follows:

$$\mathcal{L}_{class} = \lambda_1 \mathcal{L}_{\mathcal{R}} + \lambda_2 \mathcal{L}_c, \quad (S21)$$

where $\mathcal{L}_{\mathcal{R}}$ corresponds to the reconstruction loss, which is identical to the loss component utilized in the objective function for the forecasting task, and two hyperparameters λ_1, λ_2 control the contributions of the reconstruction loss and cross-entropy loss, respectively.

Section S8. Data augmentation for the classification task

In this study, we perform several data augmentation strategies on the training data of all classification datasets to achieve better generalization performance. These strategies include the following.

Noise injection: A small amount of Gaussian noise ($\sigma = 0.01$) is added to each input image with a probability of 0.1.

Translation: The images are randomly shifted by up to one pixel horizontally, vertically, or both.

Rotation: The images are subjected to a random rotation with a maximal angle of $\pm 15°$.

Flipping: We also employ random horizontal flipping with a probability of 0.5 for the input training images, except for those of the MNIST and SVHN datasets. Flipping is excluded for these digit recognition datasets since it would disrupt the semantics in the original images.

Section S9. Brief introduction to the comparative time-series forecasting algorithms in this study

In this study, we compare the OCNS with the following 7 time series forecasting algorithms: Informer [19], LogTrans [23], Reformer [24], GRU [25], LSTMa [26], LSTNet [27], and Time-LLM [28].

1. GRU. A gated recurrent unit (GRU) [25] is a type of recurrent neural network (RNN) [29] that is frequently employed in natural language processing (NLP) and time-series forecasting tasks. Similar to long short-term memory (LSTM) [30], a GRU is

designed to capture long-term dependencies and address the vanishing gradient problem that is inherent in the vanilla RNN. GRU incorporates a gating mechanism to input or forget specific features selectively. Notably, the GRU lacks a context vector or output gate, leading to a reduced parameter count and facilitating a simpler training process than that of LSTM.

2. LSTMa. Based on the encoder-decoder framework, LSTMa [26] is a neural machine translation algorithm designed to autonomously identify parts of a source sentence that are relevant to predicting a target word. This is achieved through the integration of an attention mechanism in the decoder. LSTMa seamlessly lends itself to time-series forecasting, using its encoder to process a sequence of historical time-series values and its decoder to generate a forecast of future time-series values. We use the LSTMa implementation from https://pytorch.org/tutorials/intermediate/seq2seq_translation_tutorial.html#attention-decoder.

3. LSTNet. Through the combination of a convolutional neural network (CNN) and an RNN, LSTNet [27] excels at capturing both the short-term local dependency patterns and long-term trends in multivariate time-series forecasting tasks. The traditional autoregressive model is utilized in LSTNet to solve the scale insensitivity problem faced by neural network models. We download the source code of LSTNet from https://github.com/laiguokun/LSTNet.

4. LogTrans. LogTrans [23] has emerged as a transformer-based [17] method for addressing time-series forecasting problems. Leveraging convolutional self-attention and the LogSparse transformer, LogTrans can solve the locality agnosticism and memory bottleneck issues encountered in canonical transformers, respectively. We download the source code of LogTrans from https://github.com/AIStream-Peelout/flow-forecast.

5. Reformer. Reformer [24] showcases performance comparable to that of transformer models while possessing significantly enhanced memory efficiency and processing speed for long sequences. This improvement is achieved through the introduction of locality-sensitive hashing and the incorporation of reversible residual

layers, which are aimed at optimizing the efficiency of transformers when handling long sequences. The implementation from https://github.com/lucidrains/reformer-pytorch is downloaded to conduct a performance comparison with the OCNS.

6. Informer. With three distinctive characteristics: ProbSparse self-attention, self-attention distillation, and generative style decoding, Informer [19] was purposefully crafted to address the significant challenges encountered by transformers in long sequence forecasting tasks. We download the source code of Informer from https://github.com/zhouhaoyi/Informer2020.

7. Time-LLM. By employing reprogramming of time series with prototypes and Prompt-as-Prefix, Time-LLM [28] can repurpose large language models (LLMs) for time series forecasting while keeping the backbone language models intact. In this study, two LLM backbones, GPT2 and LLAMA-7B, are employed in our experiments, denoted as Time-LLM^{a} and Time-LLM^{b}, respectively. The implementation from https://github.com/KimMeen/Time-LLM is downloaded to conduct a performance comparison with the OCNS.

8. NODE. Instead of discrete layers, neural ordinary differential equation (NODE) [31] models parameterize the derivatives of hidden states, which are then integrated over time using an ODE solver. This continuous evolution allows adaptive computation and constant memory usage, offering precise control over the trade-off between accuracy and computational cost. We download the source code of NODE from https://github.com/rtqichen/torchdiffeq.

9. SINDy AE. Sparse identification of nonlinear dynamics autoencoder (SINDy AE) [32] combines SINDy [33] with deep autoencoders to discover governing equations and corresponding coordinate systems from high-dimensional data. This approach maps the data to a lower-dimensional latent space and then identifies sparse dynamical models, balancing model simplicity and predictive accuracy. It is particularly effective for uncovering interpretable models from complex datasets where the underlying dynamics are unknown. The implementation from https://github.com/kpchamp/SindyAutoencoders is downloaded to conduct a performance comparison with the OCNS.

Section S10. Model efficiency

We comprehensively compared the computational efficiency of OCNS, OCNS* (OCNS with the "sub-time" strategy, see Section S1), and ten other current benchmark forecasting methods (Section S9). All results were recorded using a batch size of 30. In Fig. S19, we evaluate efficiency on four representative datasets (Lorenz, WTH_2, $ETTh_1$, and $ETTh_2$). Due to the channel independence strategy of Time-LLM, where each time series variate is treated as an independent channel and trained with a shared backbone, using a batch size of 30 for multivariate forecasting results in out-of-memory errors for both Time-LLM[a] and Time-LLM[b]. Consequently, the results of Time-LLM[a] and Time-LLM[b] are not included in Fig. S19.

In summary, the original OCNS outperforms other methods in terms of forecasting accuracy, but its computational efficiency is not as strong due to the lack of optimized CUDA operators that many other methods use to accelerate computation and optimize memory usage on GPUs. To address this, we propose an improved training strategy (OCNS+) that introduces a theoretically guaranteed "sub-time" concept into the OCN module (as detailed in Section S1). As shown in Fig. S19, OCNS+ significantly improves the training speed and reduces the memory footprint of OCNS, while maintaining the same level of predictive performance and state-of-the-art computational efficiency.

Section S11. Hyperparameter robustness

To thoroughly demonstrate the impact of hyperparameter choices on OCNS's performance, we conducted extensive experiments on the $EETh_1$ dataset, focusing on several key hyperparameters: the learning rate, the delay-embedding dimension S, the step size of the first delay K, the number of temporal delays D in the OCN module, and four loss component weights ($\lambda_1 \sim \lambda_4$) shown below:

- λ_1 and λ_2 control the relative importance of the reconstruction error and prediction error, respectively.
- λ_3 influences the consistency of delay embedding elements across different time stamps.

- λ_4 ensures the diversity among the states in the latent one-dimensional delay dynamical system z^t.

By varying these parameters, we evaluated their impact on the forecasting performance (MSE) of OCNS for the final prediction. The correlated results, illustrated in Fig. S20, demonstrate that OCNS exhibits robust performance across a wide range of hyperparameter settings. Although slight fluctuations in MSE were observed across different hyperparameter values, none of them led to a substantial change in the model's predictive performance. These results suggest that, overall, OCNS is not sensitive to variations in hyperparameter values, demonstrating its robustness.

Generally, for the delay-embedding dimension S, it is recommended that $S > 2d$, where d is the box-counting dimension of the input system. However, as illustrated in Fig. S20b, setting S to an excessively large value (e.g., $S = 3000$) does not yield substantial improvements in predictive performance. Therefore, we suggest choosing an appropriately large value for S to achieve an optimal trade-off between predictive performance and computational complexity. This approach ensures that the model captures the system's dynamics adequately while maintaining computational efficiency. Moreover, the selection of the weights for the regularization loss terms, λ_3 and λ_4, should be cautious. If these values are set too high (e.g., $\lambda_3 > 1.0$ and $\lambda_4 > 0.05$), the model may overemphasize regularization, potentially compromising its performance on reconstruction and prediction tasks.

Section S12. Convergence Analysis of OCNS

The convergence of OCNS is primarily ensured by the optimization algorithm used for training, which, in our case, is the Adam optimizer [34].

Adam Algorithm Convergence: The OCNS is trained end-to-end using the Adam optimizer, which is widely acknowledged in the deep learning community for its efficiency and robust convergence properties. The convergence of Adam optimizer has been extensively studied in the literature. Kingma and Ba [34] initially proposed Adam and proved its convergence to a stationary point given a bounded gradient. Subsequent

works, such as Reddi et al. [35] and Zou et al. [36], provided refined analyses and additional convergence guarantees. Chen et al. [37] extended these results to a broader class of non-convex optimization problems

Empirical Evidence of Convergence: To demonstrate the convergence of OCNS in practice, we have conducted extensive experiments across various datasets, with convergence curves presented in Fig. S21. The plot shows the training loss over epochs and illustrates that it stabilizes within 80 epochs across different datasets and scenarios. This highlights the model's consistent convergence and supports OCNS's suitability for various applications. A mathematical proof of convergence will be explored in our future work.

Section S13. Pseudocodes of OCNS

In this section, we provide the detailed pseudocodes of the proposed OCNS, outlining its computational steps and logic structure to enhance clarity and guide readers through the key steps of our algorithm:

Algorithm 1 OCNS - Overall Architecture

Input: Input $X = \left[\boldsymbol{X}^{t+1}, \boldsymbol{X}^{t+2}, \ldots, \boldsymbol{X}^{t+m}\right] \in \mathbb{R}^{n\times m}$; variate number n; length of known time series m; predicted horizon l; delay-embedding dimension S; initial temporal states length L; input weight $A \in \mathbb{R}^{L\times n}$; output weight $B \in \mathbb{R}^{n\times S}$. ▷ A and B trained using Adam optimizer

for i **in** $\{1, \ldots, m\}$ **do** ▷ Encoding process generates latent delay vectors
  $\boldsymbol{J}^{t+i} = A\boldsymbol{X}^{t+i}$ ▷ Temporal input stream $\boldsymbol{J}^{t+i} \in \mathbb{R}^L$
  for j **in** $\{0, \ldots, l\}$ **do**
    $\boldsymbol{Z}^{t+i+j} = \boldsymbol{\Phi}(\boldsymbol{J}^{t+i},\, t+i+j)$ ▷ Call OCN module to generate latent temporal delay vector $\boldsymbol{Z}^{t+i+j} \in \mathbb{R}^S$
  end for
end for
▷ Extract delay vectors $\left[\boldsymbol{Z}^{t+1}, \ldots, \boldsymbol{Z}^{t+m}, \boldsymbol{Z}^{t+m+1}, \ldots, \boldsymbol{Z}^{t+m+l}\right] \in \mathbb{R}^{S\times(m+l)}$ corresponding to the known and to be predicted time steps.
for i **in** $\{1, \ldots, m+l\}$ **do** ▷ Decoding process generates states of the original system
  $\hat{\boldsymbol{X}}^{t+m+i} = B\boldsymbol{Z}^{t+m+i}$
end for
return $\left[\hat{\boldsymbol{X}}^{t+1}, \ldots, \hat{\boldsymbol{X}}^{t+m}\right] \in \mathbb{R}^{n\times m}$, $\left[\hat{\boldsymbol{X}}^{t+m+1}, \ldots, \hat{\boldsymbol{X}}^{t+m+l}\right] \in \mathbb{R}^{n\times l}$ ▷ Return the reconstruction and prediction results

Algorithm 2 One-core-neuron (OCN) Module $\boldsymbol{\Phi}$ Algorithm Flow

Input: Step size of first delay K; temporal delay number D; OCN weights $\{w_0, w_1, \ldots, w_D\}$; delay-embedding dimension S; initial temporal states length L. ▷ $\{w_0, w_1, \ldots, w_D\}$ trained using Adam optimizer

function $\boldsymbol{\Phi}(\boldsymbol{J}^t \in \mathbb{R}^L,\, t+i)$
  Initialize an empty list $\mathcal{Z} \leftarrow []$ ▷ Store latent temporal states
  $z^{t-S-L+1}, z^{t-S-L+2}, \ldots, z^{t-S} \leftarrow [j_1^t, j_2^t, \ldots, j_L^t] = \boldsymbol{J}^t$ ▷ Initialize latent temporal states from $\boldsymbol{J}^t$ components
  for $p = 1$ to $S+i$ **do**
    $z^{t-S+p} = \mathsf{activation}(w_0 + \sum_{j=1}^{D} w_j z^{t-S+p-(K+j-1)})$ ▷ Update latent temporal states by the delay difference equation
    Append new element to $\mathcal{Z}$: $\mathcal{Z}.\mathsf{append}(z^{t-S+p})$
  end for
  $\boldsymbol{Z}^{t+i} \leftarrow \mathcal{Z}[i : S+i]$ ▷ Elements from index i to $S+i$, $\boldsymbol{Z}^{t+i} \in \mathbb{R}^S$
  return $\boldsymbol{Z}^{t+i}$ ▷ Return latent temporal delay vector corresponding to time step $t+i$
end function

Section S14. Comparative Analysis of OCNS and time-delayed reservoir computing (RC) model

The time-delayed RC model [38] offers an innovative approach by introducing time delays only in the network's output layer, which allows it to maintain the performance of a larger-scale RC while significantly reducing the physical dimensions of the reservoir network. Therefore, it offers flexible memory capacity, enabling it to tackle more complex dynamic reconstruction tasks that a standard RC of the same size cannot

easily handle. In this work, the OCNS consists of a single-neuron recurrent neural network (RNN) with two linear layers. It shares similarities with time-delayed RC, particularly in configurations with reduced neurons and delay embedding scheme, both featuring evolution dynamics and linear time-delayed readout.

However, as summarized in Table S6, OCNS and time-delayed RC differ notably in terms of their dynamic evolution processes and training parameters:

- **Dynamic Evolution Process**

OCNS is an autonomous system, whereas RC operates as a non-autonomous system. The details are explained below:

- In OCNS, the input vector $\boldsymbol{X}^t$ is multiplied by a mask (the input matrix A), and is then fed into the OCN as the initial temporal states. Subsequently, the neuron state at time t is determined exclusively by its historical neuron states at D time delays, i.e., $t-K, t-(K+1), \dots, t-(K-1+D)$, through a driven function that incorporates these historical states. The driven function is defined as:

$$z^t = \sigma\left(w_0 + \sum_{j=1}^{D} w_j z^{t-(K-1+j)}\right),$$

where σ is a nonlinear function, z^t is the one-core-neuron state at instant t, $W = \{w_j \mid j = 0, 1, \dots, D\}$ is the weight set. This driving equation constructs an autonomous delay dynamical system (difference equation).

- In RC, the neuron states at time t depend on their states at the previous time step and the input vector $\boldsymbol{X}^t$. The driven function is denoted by

$$\boldsymbol{r}^t = \alpha \boldsymbol{r}^{t-1} + (1-\alpha)\sigma(W_{in}\boldsymbol{X}^t + W_{res}\boldsymbol{r}^{t-1} + \boldsymbol{b}),$$

where σ is an activate function, W_{in} is the input matrix, adjacency matrix W_{res} represents internal connections within the reservoir, and $\boldsymbol{r}^t$ is the reservoir state at time stamp t. Therefore, the reservoir network is a non-autonomous system.

- **Training parameters**

- In RC, the input matrix W_{in}, adjacency matrix W_{res} and bias vector $\boldsymbol{b}$ are

all generated randomly, whereas only the readout weights (W_{out}) are trained with a simple learning algorithm such as linear regression.

- In OCNS, the input matrix A, the weight set W and the output matrix B are optimized through backpropagation-based algorithms, such as stochastic gradient descent (SGD).

In summary, OCNS fundamentally diverges in both its dynamic evolution and training strategies. The time-delayed RC realizes system reconstruction with remarkable efficiency by introducing time delays only in the network’s output layer with linear training, whereas OCNS leverages the advantages of both autonomous evolution (delay dynamical system) and delay embedding schemes, while also training parameters with both linear decoding and nonlinear forward propagation. This integrated approach enhances the ability of OCNS to comprehensively capture the nonlinear characteristics of original systems.

SI Reference

1. Sauer T, Yorke JA, Casdagli M. Embedology. *Journal of statistical Physics* 1991;**65**:579–616.

2. Takens F. Detecting strange attractors in turbulence. *Dynamical Systems and Turbulence, Warwick 1980*. Springer, 1981, 366–81.

3. Ma H, Leng S, Aihara K *et al.* Randomly distributed embedding making short-term high-dimensional data predictable. *Proc Natl Acad Sci USA* 2018;**115**:E9994–10002.

4. Duan X-Y, Ying X, Leng S-Y *et al.* Embedding theory of reservoir computing and reducing reservoir network using time delays. *Phys Rev Research* 2023;**5**:L022041.

5. Erneux T. *Applied Delay Differential Equations*. Springer Science & Business Media, 2009.

6. Atay FM. *Complex Time-Delay Systems: Theory and Applications*. Springer, 2010.

7. Michiels W, Niculescu S-I. *Stability, Control, and Computation for Time-Delay Systems: An Eigenvalue-Based Approach*. SIAM, 2014.

8. Casdagli M. Nonlinear prediction of chaotic time series. *Physica D: Nonlinear Phenomena* 1989;**35**:335–56.

9. Radford A, Wu J, Child R *et al.* Language Models are Unsupervised Multitask Learners.

10. Touvron H, Lavril T, Izacard G *et al.* LLaMA: Open and Efficient Foundation Language Models. 2023.

11. Deyle ER, Sugihara G. Generalized theorems for nonlinear state space reconstruction. *PLoS One* 2011;**6**.

12. Appeltant L, Soriano MC, Van der Sande G *et al.* Information processing using a single dynamical node as complex system. *Nat Commun* 2011;**2**:468.

13. Zhu Q, Guo Y, Lin W. Neural delay differential equations. *arXiv preprint arXiv:210210801* 2021.

14. Larger L, Baylón-Fuentes A, Martinenghi R *et al.* High-speed photonic reservoir computing using a time-delay-based architecture: Million words per second classification. *Physical Review X* 2017;**7**:011015.

15. Stelzer F, Röhm A, Vicente R *et al.* Deep neural networks using a single neuron: folded-in-time architecture using feedback-modulated delay loops. *Nat Commun* 2021;**12**:5164.

16. Bai S, Kolter JZ, Koltun V. An Empirical Evaluation of Generic Convolutional and

Recurrent Networks for Sequence Modeling. *arXiv:180301271 [cs]* 2018.

17. Vaswani A, Shazeer N, Parmar N *et al.* Attention is all you need. *Advances in Neural Information Processing Systems*. 2017, 5998–6008.

18. Curry JH. A generalized Lorenz system. *Communications in Mathematical Physics* 1978;**60**:193–204.

19. Zhou H, Zhang S, Peng J *et al.* Informer: Beyond Efficient Transformer for Long Sequence Time-Series Forecasting. 2021.

20. Trindade A. Electricity Load Diagrams 2011-2014. *UCI Machine Learning Repository* 2015, DOI: doi.org/10.24432/C58C86.

21. Su Y, Zhao Y, Niu C *et al.* Robust Anomaly Detection for Multivariate Time Series through Stochastic Recurrent Neural Network. *Proceedings of the 25th ACM SIGKDD International Conference on Knowledge Discovery & Data Mining*. Anchorage AK USA: ACM, 2019, 2828–37.

22. Zhang Y, Yang Q. A survey on multi-task learning. *IEEE Transactions on Knowledge and Data Engineering* 2021;**34**:5586–609.

23. Li S, Jin X, Xuan Y *et al.* Enhancing the locality and breaking the memory bottleneck of transformer on time series forecasting. *Advances in Neural Information Processing Systems*. 2019, 5244–54.

24. Kitaev N, Kaiser \Lukasz, Levskaya A. Reformer: The efficient transformer. *arXiv preprint arXiv:200104451* 2020.

25. Cho K, Van Merriënboer B, Gulcehre C *et al.* Learning phrase representations using RNN encoder-decoder for statistical machine translation. *arXiv preprint arXiv:14061078* 2014.

26. Bahdanau D, Cho K, Bengio Y. Neural Machine Translation by Jointly Learning to Align and Translate. 2016.

27. Lai G, Chang W-C, Yang Y *et al.* Modeling Long- and Short-Term Temporal Patterns with Deep Neural Networks. 2018.

28. Jin M, Wang S, Ma L *et al.* Time-LLM: Time Series Forecasting by Reprogramming Large Language Models. 2024.

29. Dupond S. A thorough review on the current advance of neural network structures. *Annual Reviews in Control* 2019;**14**:200–30.

30. Hochreiter S, Schmidhuber J. Long short-term memory. *Neural computation* 1997;**9**:1735–80.

31. Chen RTQ, Rubanova Y, Bettencourt J *et al.* Neural Ordinary Differential Equations. 2019.

32. Champion K, Lusch B, Kutz JN *et al.* Data-driven discovery of coordinates and governing equations. *Proc Natl Acad Sci USA* 2019;**116**:22445–51.

33. Brunton SL, Proctor JL, Kutz JN. Discovering governing equations from data by sparse identification of nonlinear dynamical systems. *Proc Natl Acad Sci USA* 2016;**113**:3932–7.

34. Kingma DP, Ba J. Adam: A method for stochastic optimization. *arXiv preprint arXiv:14126980* 2014.

35. Reddi SJ, Kale S, Kumar S. On the Convergence of Adam and Beyond. 2019.

36. Zou F, Shen L, Jie Z *et al.* A sufficient condition for convergences of adam and rmsprop. *Proceedings of the IEEE/CVF Conference on Computer Vision and Pattern Recognition*. 2019, 11127–35.

37. Chen X, Liu S, Sun R *et al.* On the Convergence of A Class of Adam-Type Algorithms for Non-Convex Optimization. 2019.

38. Duan X-Y, Ying X, Leng S-Y *et al.* Embedding theory of reservoir computing and reducing reservoir network using time delays. *Phys Rev Research* 2023;**5**:L022041.
